# Endothelial activating transcription factor 3 promotes angiogenesis and vascular repair in the mouse retina

**DOI:** 10.1016/j.isci.2024.111516

**Published:** 2024-12-02

**Authors:** Chihiro Ueda, Susumu Sakimoto, Masahito Yoshihara, Toru Takigawa, Akihiko Shiraki, Kaito Yamaguchi, Kosuke Shiki, Nobuhiko Shiraki, Shigetaka Kitajima, Yoshiaki Kubota, Yoko Fukushima, Kohji Nishida

**Affiliations:** 1 Department of Ophthalmology, Osaka University Graduate School of Medicine, Osaka, Japan; 2 Integrated Frontier Research for Medical Science Division, Institute for Open and Transdisciplinary Research Initiatives, Osaka University, Osaka, Japan; 3 Institute for Advanced Academic Research, Chiba University, Chiba, Japan; 4 Department of Artificial Intelligence Medicine, Graduate School of Medicine, Chiba University, Chiba, Japan; 5 Department of Biochemical Genetics, Medical Research Institute and Laboratory of Genome Structure and Regulation, School of Biomedical Science, Tokyo Medical and Dental University, Yushima, Bunkyo-ku, Tokyo, Japan; 6 Department of Anatomy, Keio University School of Medicine, Tokyo, Japan; 7 Premium Research Institute for Human Metaverse Medicine (WPI-PRIMe), Osaka University, Osaka, Japan

**Keywords:** Ophthalmology, Vascular remodeling, Transcriptomics

## Abstract

Ischemia and pathological angiogenesis in retinal vascular diseases cause serious vision-related problems. However, the transcriptional regulators of vascular repair remain unidentified. Thus, the factors and mechanisms involved in angiogenesis must be elucidated to develop approaches for restoring normal blood vessels. Here, we investigated the effects of the stress response activating transcription factor 3 (*ATF3*) on angiogenesis and vascular regeneration *in vitro* and *in vivo*. *ATF3* was expressed specifically in retinal vascular endothelial cells (ECs) during vascular development. Vascular endothelial growth factor stimulation upregulated *ATF3* expression in cultured ECs. The downregulated *ATF3* expression in ECs caused the deterioration of vascular network formation *in vitro* and *in vivo*. Moreover, *ATF3* deletion in a model of oxygen-induced retinopathy inhibited retinal vascular repair but not pathological neovascularization. Transcriptome analysis confirmed that high *ATF3* expression upregulated the expression of angiogenesis-related genes in ECs. *ATF3* may aid vascular repair therapy in retinal vascular diseases.

## Highlights

• *ATF3* promotes angiogenesis and vascular regeneration in retinal endothelial cells
• *ATF3* deficiency impairs vascular development and repair *in vitro* and *in vivo*
• *ATF3* is essential for vascular regeneration but not pathological neovascularization
• *ATF3* interacts with VEGF and other angiogenesis-related factors

## Graphical abstract

**Figure:**
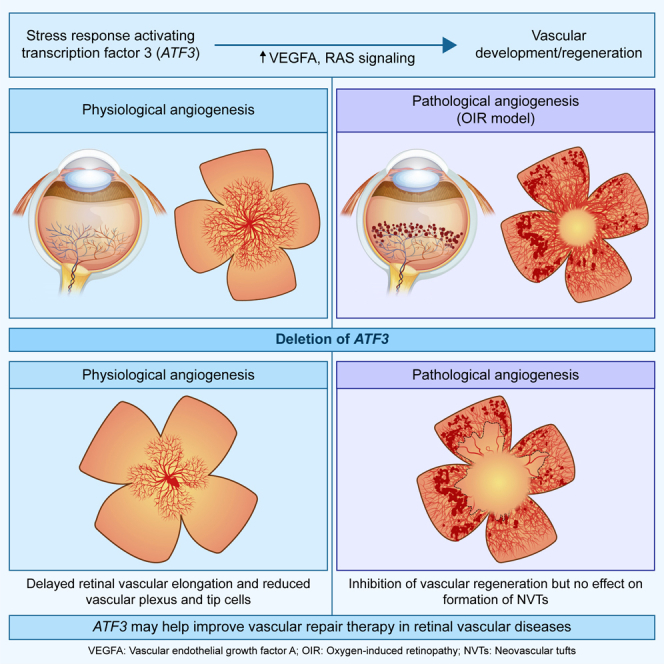

## Introduction

Retinal vascular diseases such as diabetic retinopathy, retinopathy of prematurity, and familial exudative vitreoretinopathy are the leading causes of blindness worldwide.^1^^,^^2^^,^^3^ In these diseases, during the retinal microvascular degenerative phase, the retina becomes hypoxic as peripheral vascularization becomes progressively impaired, causing retinal ischemia and leading to pathological vitreous angiogenesis.^4^ Retinal ischemia/hypoxia upregulates the expression of vascular endothelial growth factor (VEGF) through the activation of hypoxia-inducible factor 1 (*HIF-1*), leading to neovascularization.^5^^,^^6^ Clinically, the intravitreal injection of an anti-VEGF agent prevents intraocular neovascularization and improves vision quality.^7^ However, some patients are resistant to anti-VEGF therapy.^8^ In addition, anti-VEGF therapy is not a definitive solution as it does not aim to regenerate blood vessels at the site of ischemia. Therefore, understanding vessel regeneration and angiogenesis is important to develop approaches for treating ischemic diseases.

Activating transcription factor 3 (*ATF3*), which belongs to the ATF/cyclic AMP response element-binding protein family of transcription factors, plays roles in glucose and adipocyte metabolism, immune homeostasis, and oncogenesis.^9^ *ATF3* is involved in cancer cell proliferation and metastasis, but recent studies have suggested that it serves both oncogenic and tumor suppressor gene functions in regulating prostate, colon, and liver cancer. Furthermore, we previously reported that CD157-marked tissue-resident vascular endothelial stem cells have self-renewal and vascular regeneration potentials.^10^ Additionally, McDonald et al. demonstrated the important roles of *FoxM1, Myc*, and *ATF3* in vascular regeneration following physical injury.^11^ Prior to this, Wilhelm et al. had already shown that FoxO1, a FoxM1-related factor, induces Myc activity during angiogenesis in the mouse retina.^12^ Moreover, loss of the *ATF3* family gene *ATF4* in endothelial cells (ECs) inhibits exercise-induced angiogenesis.^13^ EC-specific loss of *ATF3* promotes apoptosis and decreases endothelial proliferation, leading to defects in alveolar regeneration.^14^ However, the involvement of *ATF3* in angiogenesis remains poorly understood.

Thus, this study aimed to determine whether *ATF3* modulates angiogenesis and promotes vascular repair during vascular injury. *ATF3* knockdown (*ATF3*KD) cells and endothelial-specific *ATF3* conditional knockout mice (*Atf3*iECKO) were used to confirm whether *ATF3* is crucial in retinal angiogenesis and vascular repair. This study reveals a regulatory role for *ATF3* expression in both physiological angiogenesis and vascular repair in pathological retinal models.

## Results

### Activating transcription factor 3 is localized at vascular endothelial cells in the developing mouse retina

**Figure 1:**
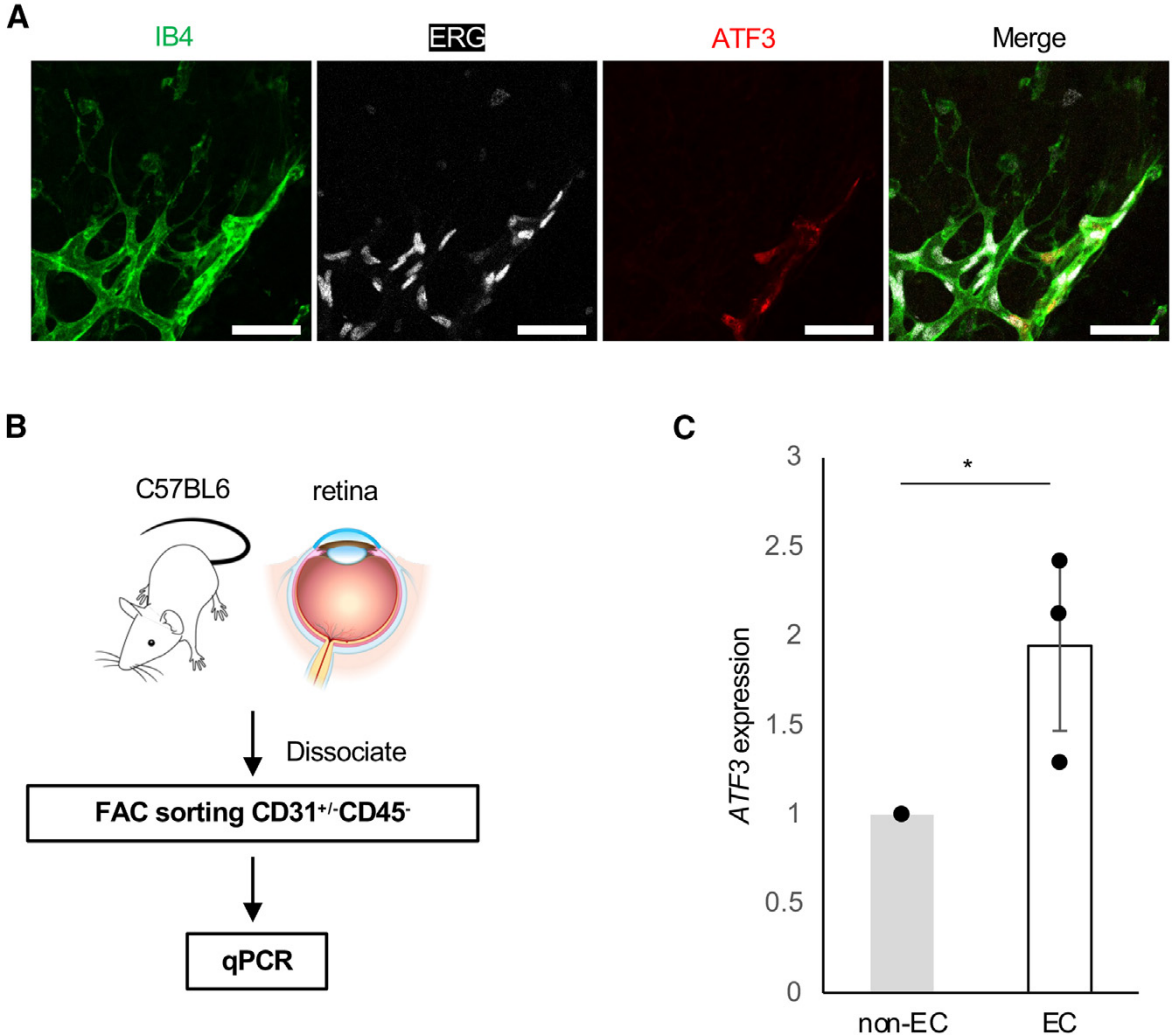
*ATF3* is localized at vascular endothelial cells (ECs) in the developing mouse retina (A) Retinal section staining of IB4 (green), ERG (magenta), and ATF3 (red) in WT mice at P5. Scale bars: 50 μm. (B) Schematic illustration of fluorescence-activated cell sorting (FACS) of mouse retinal ECs. (C) RT-qPCR analysis of mRNA in WT murine retinal non-EC (CD45-/CD31-^-^) and EC (CD45-/CD31+) at P5. Error bars represent mean ± SEM. ∗*p* < 0.05. See also Figures S1 and S2.

The retinal whole-mount was immunostained with anti-ATF3 and anti-IB4 (isolectin B4), PDGFRα, Iba1, Desmin, and PDGFRβ antibodies at postnatal day 5 (P5), during retinal vascular development, to confirm the localization of *ATF3* in the mouse retina. *ATF3* was expressed in the vascular front, corresponding to ECs (Figure 1A), but not to astrocytes, microglia, or pericytes (Figures S1A–S1D). Additionally, we confirmed that *ATF3* was expressed corresponding to ECs in frozen sections (Figure S2A). ATF3 expression was not observed in ECs of the *ATF3*iECKO mice (Figure S2B). We isolated P5 retinas from the mice, sorted them into CD45-/CD31+ cells (ECs) and CD45-/CD31-cells (non-ECs) using fluorescence-activated cell sorting (FACS), and then measured *ATF3* mRNA levels in each group (Figure 1B). Real-time quantitative PCR (RT-qPCR) results showed that *ATF3* mRNA levels were higher in the ECs than in the non-ECs (Figure 1C). These results suggest that *ATF3* expression is localized to ECs in the mouse retina.

### Deficiency of activating transcription factor 3 upregulated by VEGFA inhibits angiogenesis *in vitro*

**Figure 2:**
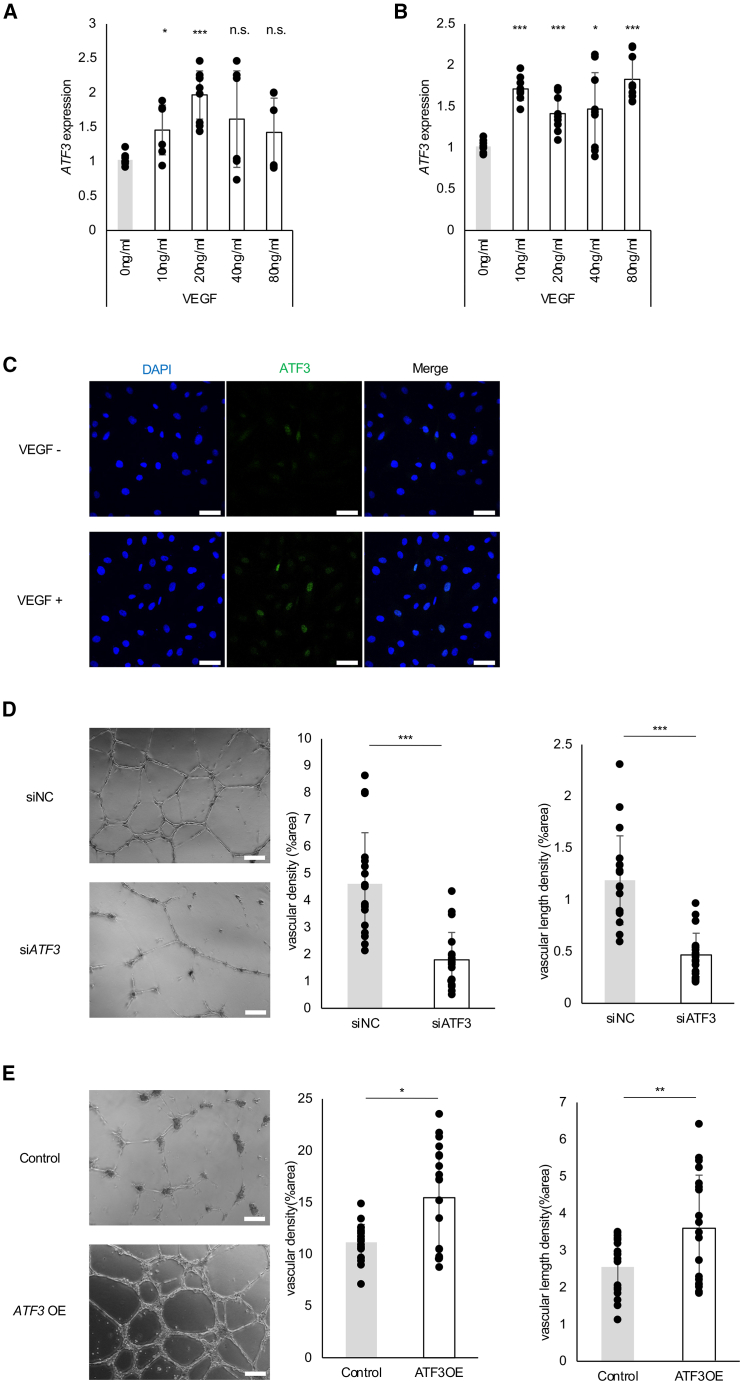
Deficiency of *ATF3* upregulated by VEGFA inhibits angiogenesis *in vitro* (A and B) RT-qPCR analysis of *ATF3* mRNA in (A) HUVECs and (B) HRMECs stimulated with VEGFA (0, 10, 20, 40, and 80 ng/mL) for 3 h after serum starvation for 6 h. (C) HRMECs were stimulated with VEGFA (20 ng/mL) for 3 h after serum starvation for 6 h and stained with ATF3 (green) and DAPI (blue). Scale bars: 50 μm. (D) Tube formation assay on Matrigel using HRMECs transfected with negative control siRNAs (siNC) or *ATF3* siRNAs (si*ATF3*). The vascular density (left) and vascular length density (right) were measured using the ImageJ Vessel Analysis plugin. (E) Tube formation assay on Matrigel using *ATF3*-overexpressed (*ATF3*OE) HUVECs infected with lentiviral vectors of human *ATF3*. The vascular density (left) and vascular length density (right) were measured using the ImageJ Vessel Analysis plugin. Scale bars: 50 μm. Error bars represent mean ± SEM. ∗*p* < 0.05, ∗∗*p* < 0.01, and ∗∗∗*p* < 0.001. See also Figures S3 and S4.

To explore the triggers of *ATF3* gene activation, we examined cultured ECs after VEGFA stimulation. RT-qPCR results confirmed that the administration of 20 ng/mL VEGFA increased *ATF3* expression in both human umbilical vein ECs (HUVECs) (Figure 2A) and human retinal microvascular ECs (HRMECs) (Figure 2B). Immunostaining of HUVECs and HRMECs also revealed an increase in *ATF3* immunofluorescence (Figure 2C). These results indicate that VEGFA stimulates *ATF3* expression. To characterize the function of *ATF3 in vitro*, we silenced *ATF3* in HRMECs by transfecting the cells with *ATF3* siRNA (si*ATF3*). RT-qPCR results confirmed that transfection with si*ATF3* reduced *ATF3* mRNA expression (Figure S3A) and decreased ATF3 protein production in HRMECs (Figure S3B). In the tube formation assay, *ATF3* knockdown suppressed vessel density compared with the negative control siRNA (siNC; Figure 2D). Moreover, we conducted the tube formation assay using *ATF3-*overexpressed (*ATF3*OE) HUVECs by infecting lentiviral vectors of human *ATF3* (Figures S4A and S4B). In the assay, *ATF3*OE HUVECs increased vessel density compared with the control (Figure 2E). Thus, these results suggest that *ATF3* expression is induced by VEGFA stimulation, and its attenuation impairs angiogenesis *in vitro*, while its overexpression promotes angiogenesis.

### Endothelial activating transcription factor 3 is required for postnatal retinal angiogenesis in mice

**Figure 3:**
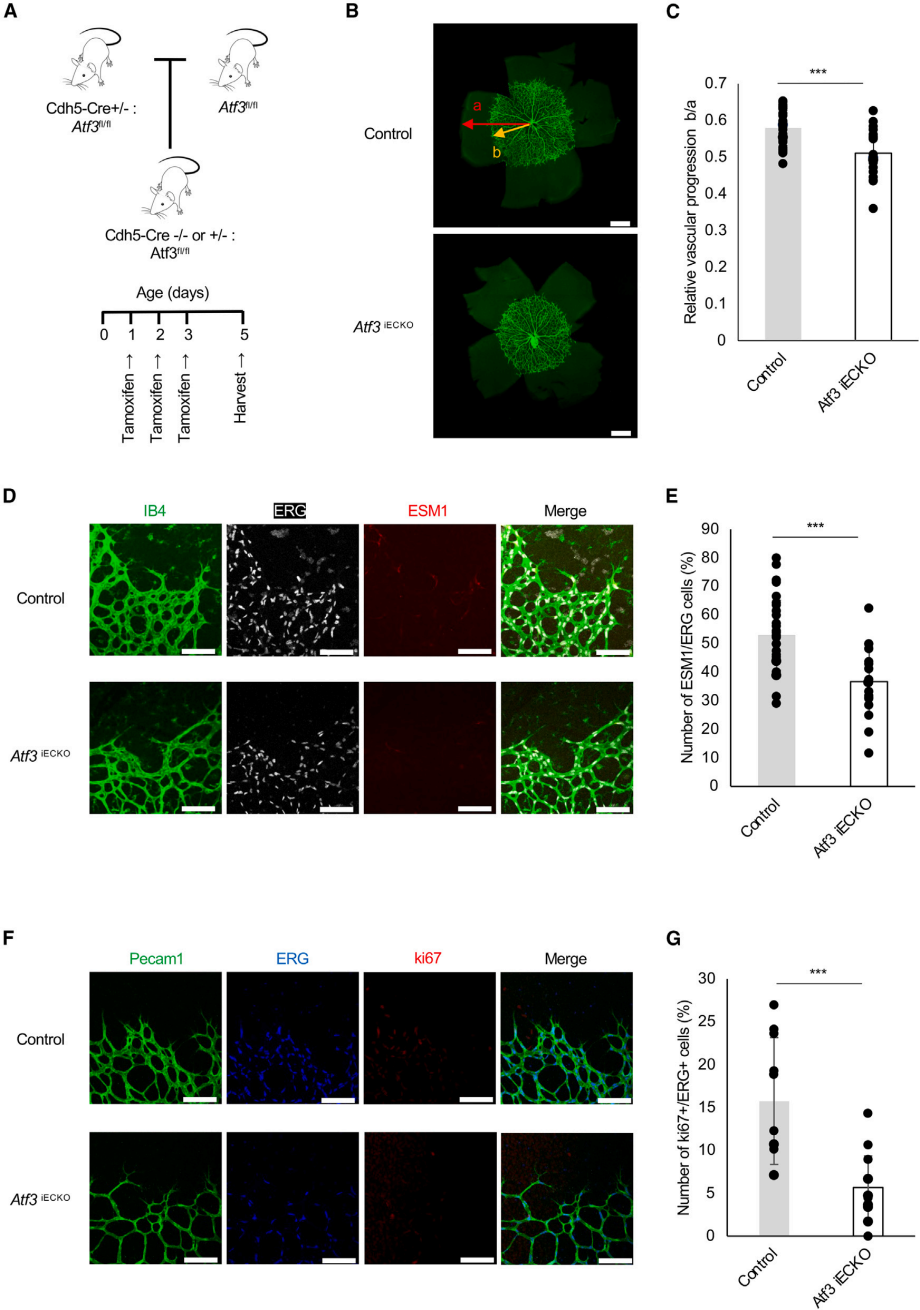
Endothelial *ATF3* is required for postnatal retinal angiogenesis in mice (A) Schematic illustration of tamoxifen administration for the generation of Cdh5-Cre *Atf3*^fl/fl^ (*Atf3*iECKO) mice. (B) Retinal whole-mount staining of PECAM1 in *Atf3*^fl/fl^ (control) and *Atf3*iECKO mice at P5. (C) Comparison of vascular progression lengths (control, *n* = 15 eyes; *Atf3*iECKO, *n* = 12 eyes). Scale bars: 500 μm. (D) Retinal whole-mount staining of IB4 (green), ESM1 (red), and ERG (white) in control and *Atf3*iECKO mice at P5. (E) Quantification of the proportion of the ESM1+ area relative to the ERG1 area in the vascular front (control, *n* = 7 eyes; *Atf3*iECKO, *n* = 8 eyes). (F) Retinal whole-mount staining of PECAM1 (green), ERG (blue), ki67 (red), and ERG (white) in control and *Atf3*iECKO mice at P5. Red color channel on the images was altered. (G) Number of ki67+/ERG+ cells/FOV in the vascular front (control, *n* = 10 eyes; *Atf3*iECKO, *n* = 13 eyes). Scale bars: 100 μm. Error bars represent mean ± SEM. ∗∗∗*p* < 0.001. See also Figure S5.

To investigate the function of *ATF3* on ECs *in vivo*, we crossed cdh5-BAC-CreERT2 mice with *Atf3* flox (*Atf3*^fl/fl^) mice. After obtaining *Atf3*iECKO mice (Figure 3A), we utilized a postnatal retinal angiogenesis model. At P5, during retinal vascular development, the *Atf3*iECKO mice showed delayed radial expansion of the vascular plexus (Figures 3B and 3C) and a significantly reduced number of vascular branch points and tip cells in the vascular front compared with the wild-type (WT) mice (Figures S5A and S5B). When these retinas were stained with ESM1, a universal marker of tip cells, the percentage of cells colored with ERG in the vascular front was reduced in the *Atf3*iECKO mice (Figures 3D and 3E). Staining with the proliferation marker ki67 also reduced the percentage of cells co-stained with ERG at the vascular front in the *Atf3*iECKO mice (Figures 3F and 3G). These results indicate that endothelial *ATF3* deletion inhibits angiogenesis during mouse retinal development.

### Activating transcription factor 3 expression is upregulated in endothelial cells of the oxygen-induced retinopathy model

**Figure 4:**
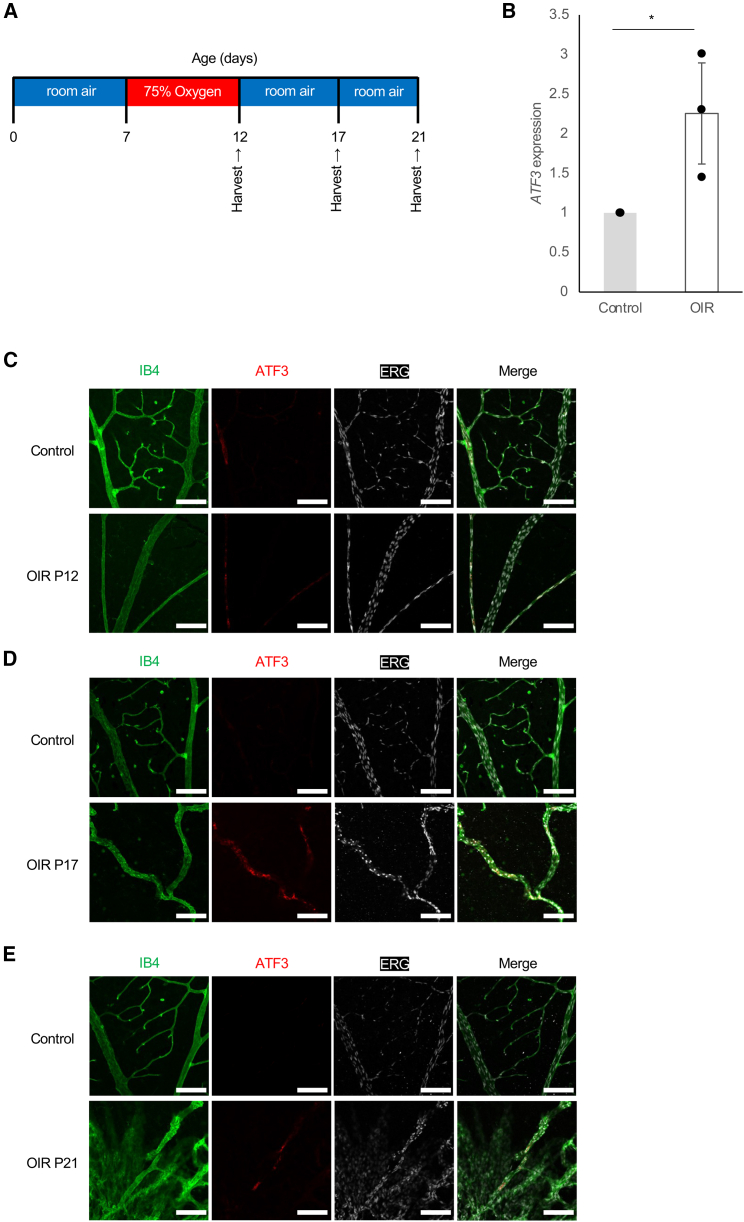
*ATF3* expression is upregulated in endothelial cells of the OIR model (A) Schematic illustration of the mouse oxygen-induced retinopathy (OIR) model. (B) RT-qPCR analysis of *ATF3* mRNA in normoxic (control) and OIR retinas at P17. Error bars represent mean ± SEM. ∗*p* < 0.05. (C) Retinal whole-mount staining of IB4 (green), ATF3 (red), and ERG (white) in control and OIR-WT mice at P12. (D) Retinal whole-mount staining of IB4 (green), ATF3 (red), and ERG (white) in control and OIR-WT mice at P17. (E) Retinal whole-mount staining of IB4 (green), ATF3 (red), and ERG (white) in control and OIR WT mice at P21. Scale bars: 100 μm. See also Figures S6 and S7.

We used an oxygen-induced retinopathy (OIR) model to investigate the role of *ATF3* in ischemic retinopathy. In this model, mice were placed in 75% oxygen (hyperoxia) at P7 to induce ischemic retinopathy by shedding blood vessels and returned to normal oxygen conditions (normoxia) at P12; neovascularization occurred and reached a maximum at P17–21 (Figure 4A).^15^^,^^16^ Neovascularization then regressed, and retinal vessels recovered at P25 (Figure 4A).^16^ We compared the *ATF3* expression in the retinal ECs of OIR WT mice with that in the retinal ECs of normoxic (control) mice at P17. Results showed that *ATF3* mRNA levels were higher in the OIR mice than in the control mice (Figure 4B). Retinal whole-mount immunostaining for *ATF3* was then performed in the OIR and control mice at P12, P17, and P21. At P12, the vessel loss phase, no difference in *ATF3* staining was found between the OIR and control mice (Figure 4C). However, the OIR mice showed increased immunoreactivity for *ATF3* at P17 (Figure 4D) and P21 (Figure 4E), the proliferative and neovascular regression phases, respectively. In frozen sections, *ATF3* was identified in ECs of mice at P7, a stage of vascular development, and of OIR mice at P17 and P21, but not at P12 (Figure S6). Unexpectedly, *ATF3* was expressed in major vessels of the retina but not in pathological vessels or neovascular tufts (NVTs) at P17 and P21 (Figure S7). These results suggest that in a mouse model of ischemic retinopathy, *ATF3* is upregulated during angiogenesis and plays a role in vascular remodeling but may not be involved in the formation of pathological vessels.

### Endothelial activating transcription factor 3 deletion inhibits vascular remodeling of oxygen-induced retinopathy retinas

**Figure 5:**
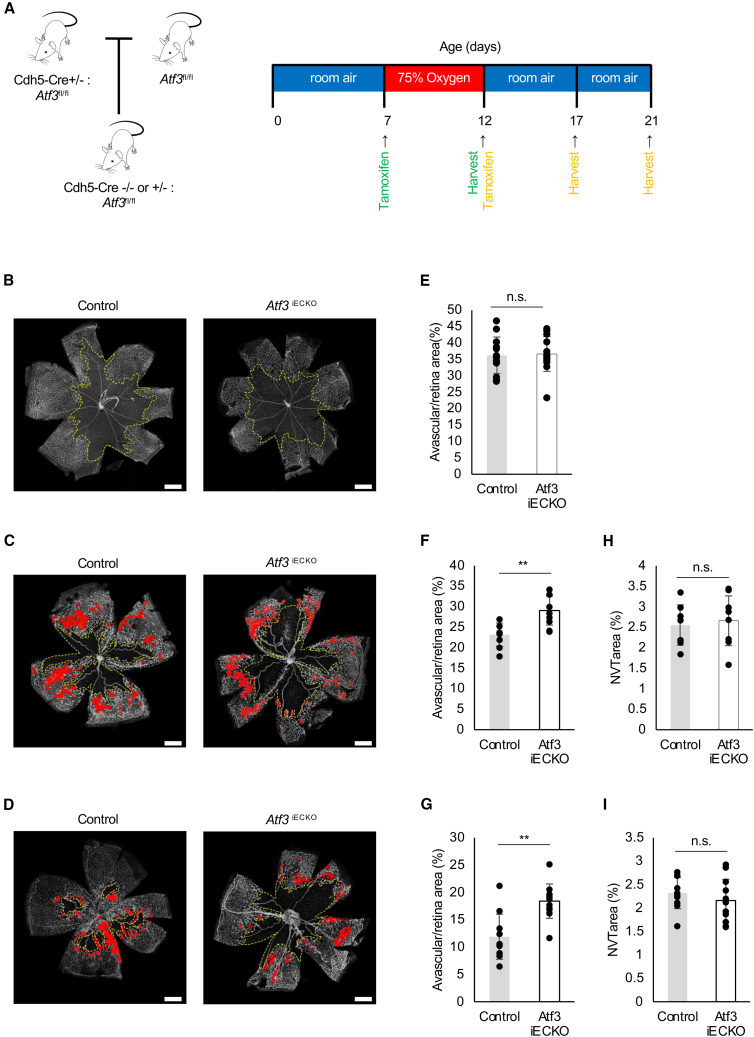
Endothelial *ATF3* deletion inhibits vascular remodeling of OIR retinas (A) Schematic illustration of the mouse OIR model and tamoxifen administration. (B–D) Retinal whole-mount staining of IB4 in *Atf3*^fl/fl^ (control) and Cdh5-Cre *Atf3*^fl/fl^ (*Atf3*iECKO) OIR mice at (B) P12, (C) P17, and (D) P21. (E–I) Quantification of the avascular area at (E) P12, (F) P17, and (G) P21, and neovascular tuft (NVT) areas at (H) P17 and (I) P21 (P12: control, *n* = 12 eyes; *Atf3*iECKO, *n* = 13 eyes) (P17: control, *n* = 7 eyes; *Atf3*iECKO, *n* = 8 eyes) (P21: control, *n* = 10 eyes; *Atf3*iECKO, *n* = 11 eyes). Scale bars: 500 μm. Error bars represent mean ± SEM. ∗∗*p* < 0.01.

To investigate the effect of *ATF3* deficiency on the retinas of OIR mice, we prepared *Atf3*iECKO mice combined with the OIR model (Figure 5A) and stained retinal whole-mount for IB4 at P12 (Figure 5B), P17 (Figure 5C), and P21 (Figure 5D). No significant differences in the avascular area at P12 (Figure 5E) were found between the *Atf3*iECKO and control mice. However, the avascular area was significantly larger in the *Atf3*iECKO mice than in the control mice at P17 (Figure 5F) and P21 (Figure 5G). Moreover, no significant differences in the NVTS area at P17 (Figure 5H) and P21 (Figure 5I) were found between the *Atf3*iECKO and control mice. These results indicate that endothelial *ATF3* deletion inhibits vascular regeneration in pathological models but does not affect the formation of pathological vessels.

### Single-cell RNA sequencing of retinal vascular endothelial cells from the oxygen-induced retinopathy mice

**Figure 6:**
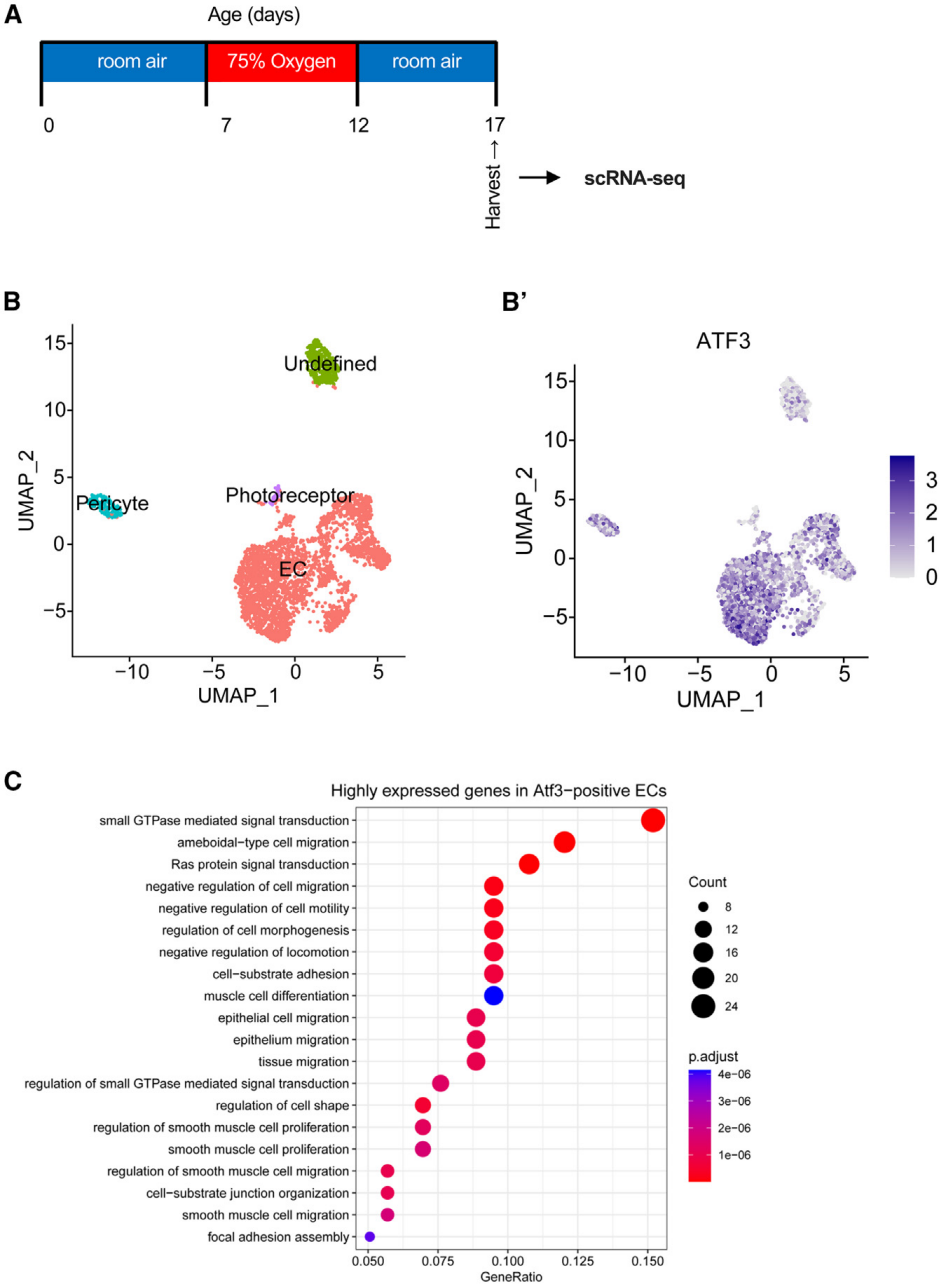
scRNA-seq of retinal vascular ECs from the OIR mice (A) Schematic illustration of the scRNA-seq mouse OIR model. (B) Uniform Manifold Projection (UMAP) of CD45-/CD31+ cells from mouse retinas in OIR at P17. (B′) UMAP colored for expression of *ATF3*. (C) Gene Ontology (GO) analysis of highly expressed genes in *ATF3*-positive ECs.

To further explore the mechanisms by which *ATF3* plays a role in a mouse model of ischemic retinopathy, we performed scRNA-seq of retinal ECs from the OIR mice. ECs from the WT mice in the OIR model were isolated at P17 and subjected to scRNA-seq (Figures 6A and 6B’; unpublished data). Gene Ontology (GO) enrichment analysis was conducted by dividing the cells in the vascular EC cluster into *ATF3*-positive and -negative cells (Figure 6C). The pathway involved in angiogenesis, Ras protein signal transduction,^17^ was upregulated in the *ATF3*-positive cells compared with the *ATF3*-negative cells.

### RNA sequencing analysis of human retinal microvascular endothelial cells transfected with activating transcription factor 3 siRNA

**Figure 7:**
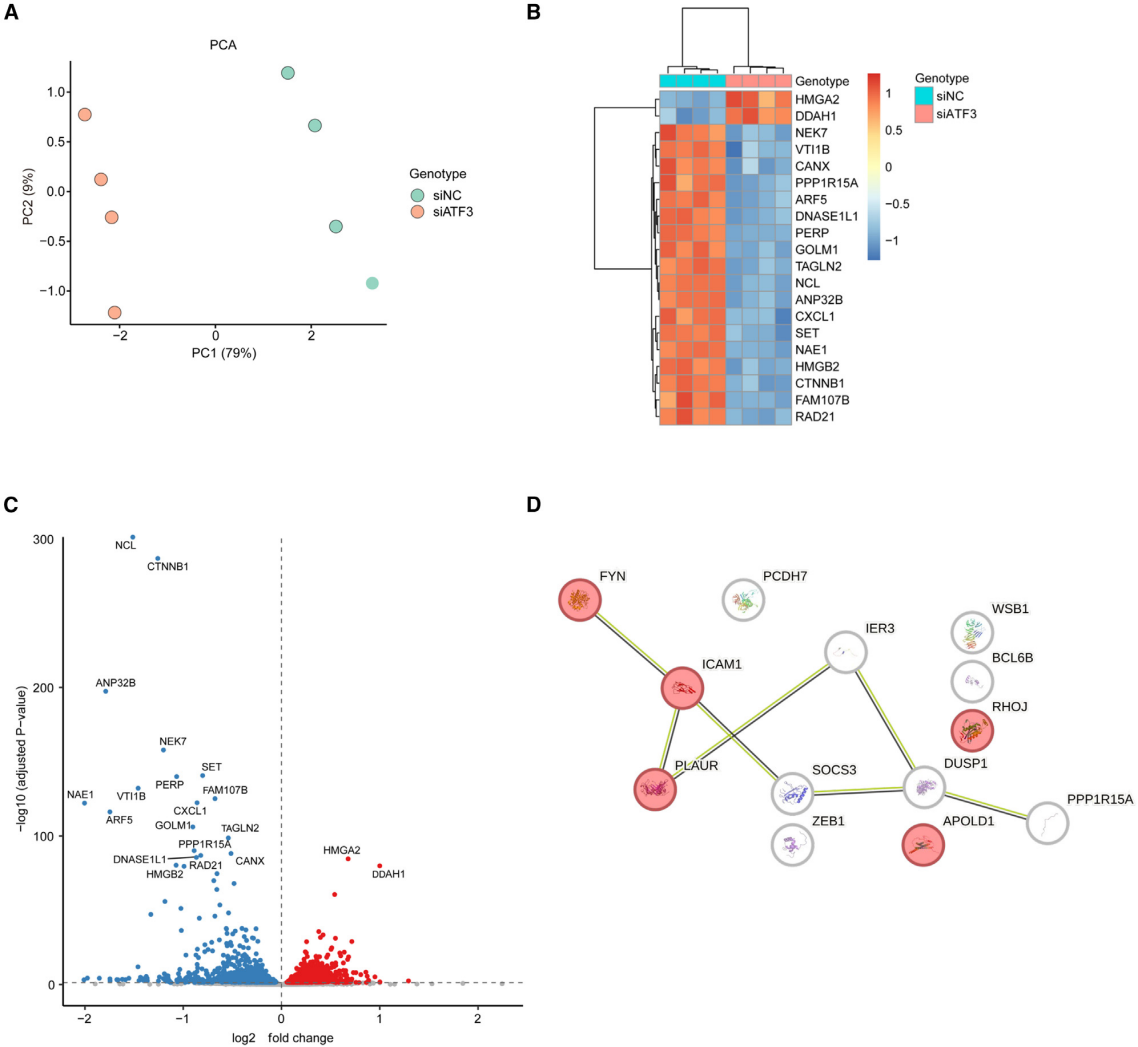
RNA-seq analysis of HRMECs transfected with negative control or *ATF3* siRNAs (A) Principal component analysis of HRMECs transfected with Silencer Select negative control siRNA (siNC) or *ATF3* siRNA (siATF3). PC1, the first principal component explains 79% of the variance; PC2, the second principal component explains 9% of the variance. *n* = 4 replicates. (B) Heatmap highlighting differentially expressed top 20 genes between *ATF3* KD HRMECs and control HRMECs. (C) Volcano plot highlighting differentially expressed top 20 genes in *ATF3* KD HRMECs compared with control HRMECs. (D) Protein–protein interaction network of factors involved in angiogenesis that are commonly downregulated in *ATF3*-negative ECs from OIR mice and *ATF3*KD HRMECs. Red-colored factors are related to the VEGFA-VEGFR2 signaling pathway.

**Figure 8:**
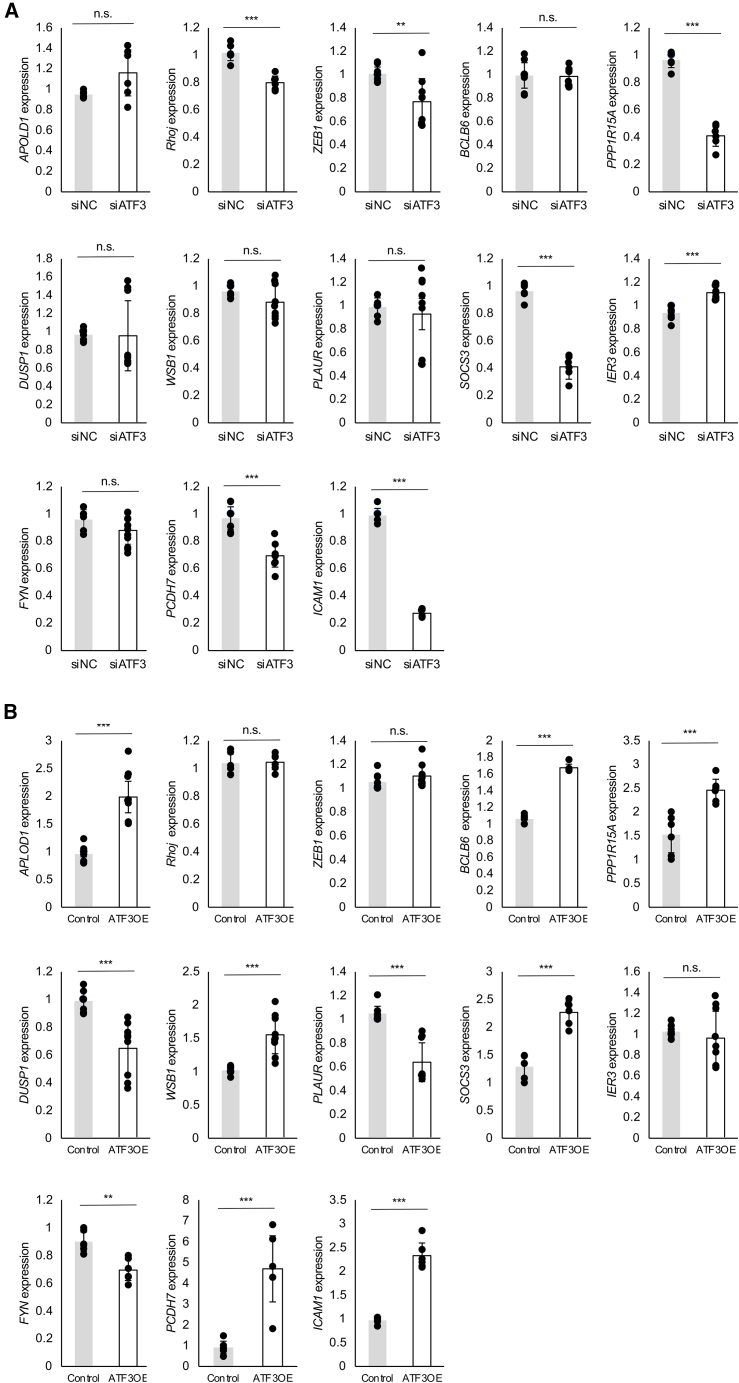
Expression of angiogenesis-related factors in *ATF3* knockdown or -overexpressed ECs (A) RT-qPCR analysis of angiogenesis-related factors in HRMECs transfected with negative control siRNAs (siNC) or *ATF3* siRNAs (si*ATF3*). (B) RT-qPCR analysis of angiogenesis-related factors in control or *ATF3-*overexpressed (*ATF3*OE) HUVECs infected with lentiviral vectors of human *ATF3*. Error bars represent mean ± SEM. ∗∗*p* < 0.01; ∗∗∗*p* < 0.001.

To determine whether the expression of angiogenesis-related genes is altered by suppression of *ATF3* expression in retinal vascular ECs, bulk RNA-seq was performed using *ATF3*KD HRMECs (Figure 7A). The top 20 genes with altered expression compared with the siNC were identified (Figures 7B and 7C), of which *CTNNB1* and *CXCL1* were reported to be associated with angiogenesis.^18^^,^^19^ These results indicate that *ATF3* is involved in regulating the expression of other angiogenesis-related factors in ECs. Thirteen of the 20 genes commonly downregulated in *ATF3*KD HRMECs and in *ATF3*-negative retinal vascular ECs from the OIR model were reported to be associated with angiogenesis (Table 1). Five of these genes were involved in the VEGFA-VEGFR2 signaling pathway (Figure 7D). Furthermore, RT-qPCR results confirmed that knockout or overexpression of *ATF3 in vitro* altered the expression levels of several angiogenesis-related factors listed in Table 1 (Figures 8A and 8B). These results suggest that *ATF3* is involved in angiogenesis in conjunction with other angiogenesis-related factors.

**Table 1:** Factors commonly downregulated in *ATF3*-negative ECs from OIR mice and *ATF3*KD HRMECs

Gene name	Other name	Role in angiogenesis	References
*APOLD1*	–	Apold1 is not required for normal angiogenesis but is involved in pathological angiogenesis.	Fan et al.^20^
*RHOJ*	–	RhoJ KO delays retinal vasculature growth, and RhoJ overexpression suppresses extraretinal vascular outgrowth in the OIR model.	Takase et al.^21^; Fukushima et al.^22^; Fukushima et al.^23^
*BCL6B*	–	BCL6B regulates retinal edema and choroidal angiogenesis via VEGF-Notch signaling modulation.	Tanaka et al.^24^
*PPP1R15A*	*GADD34*	GADD34 attenuates the phosphorylation of eIF2α (eukaryotic initiation factor 2α) and inhibits the induction of ATF4 in response to hypoxic stress.	Blais et al.^25^
*PLAUR*	–	Plaur is preferentially expressed in tip cells but is also expressed in some stem cells.	del Toro et al.^26^
*SOCS3*	–	In endothelial-specific SOCS3 KO mice, pathological angiogenesis is expanded in the OIR model, and tumor volume is increased.	Stahl et al.^27^
*FYN*	–	HRMECs treated with siRNA of FYN show inhibition of angiogenesis in a tube formation assay.	Werdich et al.^28^
*ZEB1*	–	In breast cancer, ZEB1 increases VEGFA expression and stimulates tumor growth and angiogenesis via a paracrine mechanism.	Liu et al.^29^
*DUSP1*	–	In non-small cell lung cancer, DUSP1 is expressed in cells close to the CD31 vasculature and promotes angiogenesis.	Moncho-Amor et al.^30^
*PCDH7*	–	GSEA analysis showed that angiogenesis is promoted in patients with non-small cell lung cancer.	Li et al.^31^
*WSB1*	–	In hormone receptor-negative breast cancer cell lines, downregulation of WSB-1 reduces angiogenic activity.	Poujade et al.^32^
*IER3*	–	Multiple myeloma ECs treated with siRNA or IER3 show inhibition of angiogenesis in a tube formation assay.	Ria et al.^33^
*ICAM1*	–	In rat primary brain microvascular endothelial cells (BMECs), VEGF upregulates ICAM-1 expression, resulting in BMEC migration.	Radisavljevic et al.^34^

## Discussion

In this study, we investigated whether *ATF3* promotes physiological angiogenesis and vascular repair in a mouse model of vascular injury. We found that *ATF3* was expressed in CD45-/CD31+ ECs but not in CD45-/CD31-cells in the developing mouse retina, as has been reported in other organs, such as mouse muscle, aorta, and lung.^13^^,^^14^ We demonstrated that reducing *ATF3* expression in ECs impaired vessel formation *in vitro*. Furthermore, in the endothelial-specific *Atf3*iECKO mice, vasculature development was delayed, and normal organization of the vascular network was inhibited. Interestingly, endothelial *ATF3* expression was elevated in large vessels in the OIR model but not in the control model, and it was not localized in the NVTs. Moreover, in the *Atf3*iECKO mice, the avascular area was enlarged during the proliferative and neovascular regression phases of OIR. However, no significant difference in the NVT area was found between the proliferative and neovascular regression phases of OIR. Our results suggest that *ATF3* is involved in normal angiogenesis and in angiogenesis during recovery from ischemic retinopathy but not in pathological angiogenesis.

In the OIR model, from P7 to P12, hyperoxia induces the regression of normal vasculature.^16^ After P12, the return to room air from hyperoxia induces the growth of NVTs, which peaks at P17–21 (OIR).^15^^,^^16^ The area of the vascular obliteration peaks at P12 and subsequently decreases and resolves at approximately P25.^16^ Consequently, the OIR model demonstrated a series of vascular occlusion repairs in the retina. Vascular obliteration in the *Atf3*iECKO mice at P12 was not different from that in the control but increased at P17 and P21. Our data suggest that *ATF3* is a key factor for proper regeneration of the retinal vasculature in an ischemic retinopathy model. Some studies have reported on the ATF family and endothelial regeneration in other disease models.^11^^,^^13^^,^^14^ Fan et al. reported that exercise-induced physiological angiogenesis is inhibited in vascular endothelial-specific *ATF4* KO mice.^13^ In the present study, KO of *ATF3*, which belongs to the same family as *Atf4*, inhibited physiological angiogenesis during development. Using an *in vivo* aortic endothelial injury model, a previous study has reported that the activation of stress response genes, including *ATF3*, is essential for endothelial regeneration. Global deletion of *ATF3* inhibits endothelial proliferation and compromises regeneration.^11^ Thus, *ATF3* is involved in the regeneration of ECs after physical damage. This study further showed that it is involved in angiogenesis. Niethamer et al. reported that the number of pulmonary ECs is reduced in *Atf3*iECKO mice after influenza infection because of decreased proliferation and increased apoptosis.^14^ In the present study, the number of tip cells and expression of proliferative markers were also reduced during normal retinal vascular development in *Atf3*iECKO mice. This result indicates that *ATF3* expression contributes to the proliferative potential of ECs during normal vascular development. Additionally, in the present study, the area of NVTs in the *Atf3*iECKO mice did not differ from that in the control mice. Okamoto et al. suggested that stimulation by reactive oxygen species may cause pathological angiogenesis via *ATF3* in microvascular disorders in diabetes mellitus.^35^ By contrast, our pathological model suggests that *ATF3* does not contribute to the formation of pathological neovascularization due to retinal ischemia.

The present study also showed that VEGFA increased the expression of *ATF3*, and previous studies have reported on VEGF and the ATF family. Oxidized phospholipids induce VEGF expression by activating the *ATF4* branch of the unfolded protein response.^36^ This study shows that oxidized sn-2 residues in lipids play a crucial role in triggering UPR/*ATF4*-dependent gene transcription and subsequently increasing VEGF levels.^36^ *ZNFTR* interacts with *ATF3* to sequester *ATF3* away from the *ZNF24* promoter, increase *ZNF24* expression, and downregulate VEGFA transcription, thereby reducing the proliferative, metastatic, and proangiogenic abilities of pancreatic cancer cells. Under hypoxic conditions, downregulation of *ZNFTR* expression decreases *ZNF24* expression via *ATF3*, resulting in the upregulation of VEGFA, which promotes pancreatic cancer progression.^37^

In scRNA-seq of retinal vascular ECs in the proliferative phase of OIR, the Ras signal transduction pathway was highly expressed in the *ATF3*-positive cells. The Ras protein induces angiogenesis through the MEK/ERK pathway,^17^ suggesting that angiogenesis may be enhanced in *ATF3*-positive cells.

In the RNA-seq analysis of *ATF3*KD ECs, among the differentially expressed genes, we focused on the downregulation of *CTNNB1* (β-catenin) and *CXCL1* because of their association with angiogenesis. The Wnt signaling pathway, which is involved in the coordinated regulation of gene expression and precise interactions between neighboring cells,^38^ influences the functions of various angiogenic factors, including VEGF, and mediates angiogenesis.^39^ Its major component, β-catenin (*CTNNB1*), also functions as a component of the cadherin complex, which regulates EC adhesion and contributes to vascular stabilization.^38^^,^^39^ Furthermore, the endothelium-specific deletion of *Ctnnb1* during retinal angiogenesis in mice significantly reduces vascular density.^18^ This phenotype was similar to that observed in the endothelium-specific *ATF3*-deficient mice in the present study. *CXCL1* is expressed and secreted by ECs, and it induces angiogenesis via the *CXCR2* receptor, ERK1/2, and EGF pathways.^19^ Thus, the reduced expression of *ATF3* in vascular ECs *in vitro* affects other angiogenesis-related factors.

Furthermore, we identified 20 genes commonly downregulated in *ATF3*-negative retinal ECs from OIR and *ATF3*KD ECs. Among these genes, *APOLD1*, *RHOJ*, *ZEB1*, *BCL6B*, *PPP1R15A*, *DUSP1*, *WSB1*, *PLAUR*, *SOCS3*, *IER3*, *FYN*, *PCDH7*, and *ICAM1* are associated with angiogenesis (Table 1). *APOLD1* is activated in ECs during growth factor stimulation and hypoxia, regulating EC proliferation.^20^ *RhoJ* KO mice show delayed vasculature growth in the developing mouse retina,^21^^,^^22^ and overexpression of *RhoJ* in OIR mice significantly reduces the number of extraretinal vessels and decreases intraretinal capillary density.^23^ *BCL6B* regulates retinal edema and choroidal angiogenesis by modulating VEGF-Notch signaling.^24^ *GADD34* (*PPP1R15A*) attenuates the phosphorylation of eIF2α (eukaryotic initiation factor 2α) and inhibits the induction of *ATF4* in response to hypoxia.^25^ *PLAUR* is preferentially expressed in tip cells but is also expressed in some stem cells.^26^ In endothelial-specific *SOCS3* KO mice, pathological angiogenesis is promoted in the OIR model, and tumor volume is increased.^27^ Tube formation assay results showed that transfecting HRMECs with *FYN* siRNA inhibits angiogenesis.^28^ Expression of *ZEB1*, *DUSP1*, and *PCDH7* promotes angiogenesis in breast^29^ and lung^30^^,^^31^ cancer. Downregulation of *WSB1* and *IER3* reduces angiogenic effects in breast cancer^32^ and myeloma.^33^ Furthermore, similar to the results of scRNA-seq and bulk RNA-seq, RT-qPCR results showed relatively lower expression levels of *RHOJ, ZEB1, PPP1R15A, SOCS3, PCDH7*, and *ICAM1* in *ATF3*KD HRMECs than in the control (Figure 8A). Among these genes, the expression of *PPP1R15A*, *SOCS3*, *PCDH7*, and *ICAM1* increased in *ATF3*OE HUVECs (Figure 8B). *PPP1R15A*, *SOCS3*, and *ICAM* correlated with each other in the protein–protein interaction network analysis of factors involved in angiogenesis (Figure 7D). We hypothesize that these genes are involved in angiogenesis downstream of the transcription factor *ATF3*. Furthermore, ICAM is one of the genes related to the VEGFA-VEGFR2 signaling pathway (Figure 7D). A previous study reported that stimulation by VEGF upregulates the expression of *ICAM-1* and promotes the migration of brain microvascular ECs in mice.^34^ *ATF3* may be responsible for angiogenesis via the VEGF pathway in correlation with ICAM1. These results support the hypothesis that changes in *ATF3* expression affect angiogenesis.

Overall, we showed that *ATF3* played an important role in the normal development of blood vessels in the mouse retina and in vascular regeneration in models of ischemic retinopathy. We also found that suppression of *ATF3* expression altered the expression of several angiogenesis-related factors, including the VEGF signaling pathway. Further validation of the pathways through which *ATF3* influences angiogenesis is required.

### Limitations of the study

This study has some limitations. First, we could not clarify why ATF3 is involved in normal angiogenesis but not in pathological angiogenesis. During angiogenesis, tip cells act as guides, and the stalk cells behind them have high proliferative activity and form blood vessels.^40^ The relationship between ATF3 and tip cells was suggested by immunostaining and the reduction of tip cells in *Atf3*iECKO mice. The involvement of ATF3 in EC proliferation was also predicted by the upregulation of the Ras protein signal, which regulates EC proliferation,^41^ and the downregulation of proliferation markers in *Atf3*iECKO mice. However, ATF3 knockout had no effect on the formation of pathological blood vessels, which is inconsistent with the above results. Second, the detailed mechanism by which ATF3 directly influences angiogenesis remains unclear. We performed RT-qPCR (Figure 8) using the factor extracted in Figure 7D to clarify the potential target genes and signaling pathways of ATF3. Using the factors and pathways suggested to be associated with ATF3 in this study as bases, we need further research to clarify the mechanism by which ATF3 regulates angiogenesis and elucidate why ATF3 is not involved in pathological vessel growth. Finally, because it is difficult to distinguish the sex of immature mice, the influence of sex on the results remains unclear.

## Resource availability

### Lead contact

Requests for further information and resources should be directed to and will be fulfilled by the lead contact, Susumu Sakimoto (susumu.sakimoto@ophthal.med.osaka-u.ac.jp).

### Materials availability

This study did not generate new unique reagents.

### Data and code availability

• Data: Bulk RNA-seq data have been deposited at GEO and are publicly available as of the date of publication. Accession numbers are listed in the key resources table.
• Code: This study does not report the original code.
• Additional information: Any additional information required to reanalyze the data reported in this article is available from the lead contact upon request.

## Acknowledgments

We thank Yuki Ishikawa and Tomohiko Katayama for technical advice and Noriko Izumi for administrative support. This study was supported by the Center for Medical Research and Education of the Graduate School of Medicine, 10.13039/501100004206Osaka University, Japan. This work was supported by grants from 10.13039/100009619AMED under grant number 23gm121000 and a Grant-in-Aid for Scientific Research (21K09674) from the 10.13039/501100001691Japan Society for the Promotion of Science. This work was supported by 10.13039/501100008667SENSHIN Medical Research Foundation, the 10.13039/100008695Japan Foundation for Applied Enzymology (VBIC), Takeda Science Foundation Medical Research Grants, Charitable Trust Fund for Ophthalmic Research in Commemoration of Santen Pharmaceutical’s Founder, The Global Ophthalmology Awards Program, and Bayer Retina Award.

## Author contributions

CU, SS, and KN designed the study. MY analyzed the bulk RNA-seq and the single-cell RNA-seq data. AS, KY, KS, and NS supported the experiments. SK and YK provided mice. YF provided critical experimental equipment and reagents. CU and TT performed the experiments. CU analyzed the data. KN acquired financial support. CU and SS wrote the article. All authors have read and agreed to the article.

## Declaration of interests

The authors declare no competing interests.

## STAR★Methods

### Key resources table

**Table:** 

REAGENT or RESOURCE	SOURCE	IDENTIFIER
**Antibodies**		

Anti-mouse CD16/CD32	BD Biosciences	Cat#:553141; RRID: AB_394656
Brilliant Violet 421 anti-mouse CD31	Biolegend	Cat#: 102424; RRID: AB_2650892
FITC anti-mouse CD45	Biolegend	Cat#: 103108; RRID: AB_312972
GS-IB4 from *Griffonia simplicifolia*, Alexa Fluor 488 conjugate	Invitrogen	Cat#: I21411; RRID: AB_2314662)
Goat anti-rabbit IgG Fab Fragment	Jackson ImmunoResearch Laboratories	Cat#: 111-007-003; RRID: AB_2337925
Rat anti-CD31(PECAM1)	BD Biosciences	Cat#: 553370; RRID: AB_394816
Rabbit anti-ERG	Abcam	Cat#: ab92513; RRID: AB_2630401
Mouse anti-ATF3	Santa Cruz Biotechnology	Cat#: sc-81189; RRID: AB_2058591
Rabbit anti-ATF3	Cell Signaling Technology	Cat# 18665; RRID: AB_2827506
Rat anti-CD140α (PDGFRα)	BD Biosciences	Cat#: 558774; RRID: AB_397117
Rabbit anti-Iba1	Fujifilm Wako Co	Cat#: 019-19741; RRID: AB_839504
Rabbit anti-Desmin	Abcam	Cat#: ab15200; RRID: AB_301744
Rabbit anti-PDGFRβ	Cell Signaling Technology	Cat#: 3169; RRID: AB_2162497
Goat anti-ESM1	R&D systems	Cat#: AF1999; RRID: AB_2101810
Rabbit anti-ki67	Invitrogen	Cat#: MA5-14520; RRID: AB_10979488
Rabbit anti-ATF3	Cell Signaling Technology	Cat# 33593; RRID: AB_2799039
Mouse anti-βactin	Sigma-Aldrich	Cat#: A5441; RRID: AB_476744
Alexa Flour 488 goat anti-rat IgG	Invitrogen	Cat#: A-11006; RRID: AB_2534074
Alexa Flour 488 goat anti-mouse IgG	Invitrogen	Cat#: A-11001; RRID: AB_2534069
Alexa Flour 488 goat anti-rabbit IgG	Invitrogen	Cat#: A-11008; RRID: AB_143165
Alexa Flour 568 Donkey anti-goat IgG	Invitrogen	Cat#: A-11057; RRID: AB_2534104
Alexa Flour 594 goat anti-rat IgG	Invitrogen	Cat#: A-11007; RRID: AB_10561522
Alexa Flour 594 goat anti-mouse IgG	Invitrogen	Cat#: A-11005; RRID: AB_2534073
Alexa Flour 594 goat anti-rabbit IgG	Invitrogen	Cat#: A-11012; RRID: AB_2534079
Alexa Flour 647 goat anti-rat IgG	Invitrogen	Cat#: A-21247; RRID: AB_141778
Alexa Flour 647 goat anti-rabbit IgG	Invitrogen	Cat#: A-21244; RRID: AB_2535812
Donkey anti-rabbit IgG horseradish peroxidase (HRP)	Cytiva	Cat#: NA934; RRID: AB_772206
Sheep anti-mouse IgG horseradish peroxidase (HRP)	Cytiva	Cat#: NA931; RRID: AB_772210

**Chemicals, peptides, and recombinant proteins**		

Tamoxifen	Sigma-Aldrich	Cat#: T5648-1G
Corn oil	Sigma-Aldrich	Cat#: C8267
Dispase I	Roche	Cat#: 4942086001
Collagenase I	Worthington	Cat#: CLSS1
Collagenase Ⅱ	Worthington	Cat#: CLSS2
Fetal bovine serum (FBS)	Gibco	Cat#: 26140079
DNase I	Takara	Cat#: 2270B
Propidium iodide	Immunostep	Cat#: PI
DAPI	Biotium	Cat#: 40043
HuMedia-EG2	KURABO	Cat#: KE-2150S
CS-C Medium Kit R (w/o Serum)	Cell Systems	Cat#: CS4Z3500R
VEGF 165	PeproTech	Cat#: 100-20
Lipofectamine RNAiMAX Transfection Reagent	Invitrogen	Cat#: 13778075
RIPA Buffer	Nacalai Tesque	Cat#: 16488-34
Matrigel Matrix	CORNING	Cat#: 354234

**Critical commercial assays**		

Rneasy Micro Kit	QIAGEN	Cat#: 74004
Rneasy Mini Kit	QIAGEN	Cat#: 74106
PrimeScript RT Master Mix	Takara	Cat#: RR036
THUNDERBIRD SYBR® qPCR Mix	TOYOBO	Cat#: QPS-201
Lentiviral High Titer Packaging Mix	Takara	Cat#: 6194
*Trans*IT®-Lenti Transfection Reagent	Mirus Bio	Cat#: MIR6603
NEBNext Poly(A) mRNA Magnetic Isolation Module	New England Biolabs	Cat#: E7490
NEBNext Ultra II Directional RNA Library Prep Kit	New England Biolabs	Cat#: E7760
SPRIselect Reagent Kit	Beckman Coulter	Cat#: B23318
Chromium Single Cell 3′ Library & Gel Bead Kit v3	10x Genomics	Cat#: PN-1000092
High Sensitivity DNA Kit	Agilent Technologies	Cat#: 5067-4626

**Deposited data**		

Bulk RNA-seq datasets of HRMECs transfected with *ATF3* siRNA	Gene Expression Omnibus (GEO)	GEO: GSE249642

**Experimental models: Cell lines**		

Human umbilical vein endothelial cells (HUVECs)	KURABO	Cat#: KE-4109
Human Retinal Microvascular Endothelial Cells (HRMECs)	Cell Systems	Cat#: CSACBRI181
Lenti-X™ 293T Cell Line	Takara	Cat#: 632180

**Experimental models: Organisms/strains**		

Mouse: C57BL/6J	Japan SLC	N/A
Mouse: C57BL/6J	Charles River Laboratories	RRID: IMSR_JAX:000664
*Mouse: Atf3*^fl/fl^	Shigetaka Kitajima^42^	N/A
Mouse: Cdh5-BAC-CreERT2	Yoshiaki Kubota^43^	N/A

**Oligonucleotides**		

Stealth siRNA for human *ATF3*	Invitrogen	Cat#: 1299001
Stealth RNAi siRNA Negative Control	Invitrogen	Cat#: 12935112
Silencer Select siRNAs targeting human *ATF3*	Ambion	Cat#: 4392420
Silencer Select Negative Control No. 2 siRNA	Invitrogen	Cat#: 4390846
See Table S1 for the qPCR primers		

**Software and algorithms**		

ImageJ (v1.54)	National Institutes of Health	https://imagej.net/; RRID: SCR_003070
Photoshop (v23.5.1)	Adobe Systems	https://www.adobe.com/products/photoshop.html; RRID:SCR_014199
JMP Pro (v17.1.0)	SAS Institute	https://www.jmp.com/en_us/home.html; RRID: SCR_022199
fastp (v0.19.5)	Chen et al.^44^	https://github.com/OpenGene/fastp
STAR (v2.7.10b)	Dobin et al.^45^	https://github.com/alexdobin/STAR/releases
RSEM (v1.3.3)	Li et al.^46^	https://deweylab.github.io/RSEM/
DESeq2 (v1.38.3)	Love et al.^47^	https://www.bioconductor.org/packages/release/bioc/html/DESeq2.html
Cell Ranger v7.1.0	10x Genomics	N/A
R package SoupX (v1.6.2)	Young et al.^48^	https://github.com/constantAmateur/SoupX
Python package scrublet (v0.2.3)	Wolock et al.^49^	https://github.com/AllonKleinLab/scrublet
R package Seurat (v4.3.0)	Hao et al.^50^	https://github.com/satijalab/seurat
ClusterProfiler package (v4.6.2)	Wo et al.^51^	https://bioconductor.org/packages/release/bioc/html/clusterProfiler.html

### Experimental model and study participant details

#### Mice

The C57BL/6J mouse line was purchased from SLC and Charles River Laboratories, Japan, Inc. The *Atf3*^fl/fl^ mouse line was obtained from Shigetaka Kitajima (Tokyo Medical and Dental University, Tokyo, Japan).^42^ Cdh5-BAC-CreERT2 mice^43^ were crossed with *Atf3*^fl/fl^^42^ mice to generate endothelial-specific conditional *Atf3* knockout (*Atf3*iECKO) mice. *Atf3*^fl/fl^ or *Atf3*iECKO mice were treated with 50 μg tamoxifen (Sigma-Aldrich, Saint Louis, MO, USA) in corn oil (Sigma-Aldrich) daily from P1 to P3 and dissected at P5. In the OIR model, the mice were treated with 4 mg/25 g BW tamoxifen at P7 and dissected at P12 or treated with 4 mg/25 g BW tamoxifen at P12 and dissected at P17 and P21. All mice were housed in a controlled environment with a 12-hour light-dark cycle and had free access to food and water. Both male and female mice were included in analyses as it is difficult to distinguish the sex of immature mice, such as those at P5 or P7 corresponding to the start of the OIR model. All the animal experiments were reviewed and approved by the Institute of Experimental Animal Sciences, Faculty of Medicine, Osaka University, Japan.

#### Cell culture

HUVECs were purchased from KURABO Biomedical Business (Osaka, Japan). They were seeded at a density of 1.0 × 10^4^ cells/cm^2^ in HuMedia-EG2 medium (KURABO Biomedical Business) containing 2 % fetal bovine serum, 10 ng/mL recombinant human epidermal growth factor, 1 μg/mL hydrocortisone, 50 μg/mL gentamycin, 50 ng/mL amphotericin B, 5 ng/mL recombinant human basic fibroblast growth factor , and 10 μg/mL heparin. HRMECs were purchased from Cell Systems (Kirkland, WA, USA) were seeded at a density of 1.0 × 10^4^ cells/cm^2^ on Attachment Factor (Cell Systems)-coated dishes (Falcon) in CS-C medium (Cell Systems) with Defined Cell Boost (Cell Systems) and 10% FBS. After 6 h of serum starvation in 0.5% FBS, the cells were stimulated with 20 ng/mL VEGF 165 (PeproTech, London, UK). The cells were cultured at 37°C in 5% CO_2._ The cells were authenticated by the suppliers, who guaranteed them to be negative for mycoplasma.

### Method details

#### Flow cytometry

Mouse retinas were incubated in 2.1 units/mL dispase I (Roche, Basel, Switzerland) and 1% collagenase I and II (Worthington, Lakewood, NJ, USA) in 4% fetal bovine serum containing 0.05 units/mL Dnase I (Takara, Shiga, Japan). Retinas were disrupted using an 18G needle and dissociated into single cells using a 40 μM cell strainer (Falcon, Franklin Lakes, NJ, USA). The cells were blocked with anti-mouse CD16/CD32 (Fc Blocker) (BD Biosciences, San Jose, CA, USA) and stained with Brilliant Violet 421 anti-mouse CD31 (Biolegend, San Diego, CA, USA) and FITC anti-mouse CD45 antibodies (BioLegend). Dead cells were stained with propidium iodide (Immunostep, Salamanca, Spain) and excluded. Cells were sorted using a BD FACS Melody™ Cell Sorter (BD Biosciences).

#### Fluorescence and light microscopy

For whole-mount retinas, the eyes were enucleated; dissected in phosphate-buffered saline (PBS); fixed for 2 h in 4% paraformaldehyde (PFA) in PBS; washed in 40%, 70%, and 100% methanol; and then stored overnight at −30°C in 100% methanol. The next day, the retinas were washed in 70% and 40% methanol and incubated overnight in blocking buffer (2% skim milk, 5% NGS, 1% bovine serum albumin (BSA), and 0.1% Tween20 in PBS). For retinal cryosections, the eyes were enucleated, dissected in PBS, and then fixed overnight in 4% PFA in PBS. The next day, the retinas were cryoprotected in 30% sucrose and frozen, sectioned at a thickness of 10 μm with a cryostat, and then incubated for 20 min in blocking buffer (2% skim milk, 5% NGS, 1% BSA, and 0.1% Tween 20 in PBS). The retinal wholemounts and cryosections were incubated overnight at 4°C with primary antibodies diluted in blocking buffer and then incubated overnight at 4°C with appropriate secondary antibodies. For staining with isolectin GS-IB4 from *Griffonia simplicifolia*, Alexa Fluor 488 conjugate (Invitrogen, Carlsbad, CA, USA), the retinas were fixed at room temperature and then incubated in blocking buffer (1% BSA and 0.3% Triton X-100 in PBS).^52^ Then, they were blocked overnight with goat anti-rabbit IgG Fab Fragment (Jackson ImmunoResearch Laboratories, West Grove, PA, USA) for simultaneous staining of anti-rabbit ERG and ki67. Fluorescein images were acquired using a Zeiss LSM 710 confocal microscope (Carl Zeiss, Oberkochen, Germany). The radial length of the retinal vessel was measured as the distance from the optic disc to the front of the vascular network divided by the distance from the optic disc to the retinal margin. The numbers of ESM1+ ERG+ cells, ki67+ ERG+ cells, branch points, and tip cells were measured manually and normalized per mm^2^ of retina tissue. In the OIR model, the avascular area and surface area of NVTs were measured and divided by the total retinal area. All images were analyzed using ImageJ software (v1.54) (National Institutes of Health, Bethesda, MD, USA) or Adobe Photoshop software version (v23.5.1) (Adobe Systems Incorporated, Seattle, WA, USA). For immunostaining of cells, they were fixed for 10 min in 4% PFA incubated in blocking buffer (2% skim milk, 5% NGS, 1% bovine serum albumin, and 0.1% Tween20 in PBS) for 2 h and then incubated overnight at 4°C with primary antibodies diluted in blocking buffer. The following day, the retinas were incubated with the appropriate secondary antibodies for 2 h. Fluorescein images were acquired using an Olympus SV3000 confocal microscope (Olympus, Tokyo, Japan).

Antibodies for staining were anti-PECAM1 (1/200, BD Biosciences), anti-ERG (1/200, Abcam, Cambridge, UK), anti-ATF3 (1/200, Santa Cruz Biotechnology, Santa Cruz, CA, USA), anti-ATF3 (1/100, Cell Signaling Technology, Danvers, MA, USA), anti-CD140α (PDGFRα) (1/200, BD Biosciences), anti-Iba1 (1/200, Fujifilm Wako Co., Osaka, Japan), anti-Desmin (1/200, Abcam), anti-PDGFRβ (1/200, Cell Signaling Technology), anti-ESM1 (1/200, R&D systems, Minneapolis, MN, USA), anti-ki67 (1/200, Invitrogen), DAPI (1/1000, Biotium, Hayward, CA, USA), and AlexaFluor® 488-conjugated isolectin GS-IB4 (40 μg/mL, Invitrogen). The secondary antibody was Alexa Fluor 488/568/594/647-conjugated IgG (1/500, Invitrogen).

#### OIR model

The OIR model mimics retinopathy of prematurity by inducing ischemic retinopathy through vascular regression in a hyperoxic environment, followed by pathological neovascularization upon returning to normal oxygen conditions. The mouse pups (male and female) and their nursing mothers were placed in 75% oxygen (hyperoxia) from P7 to P12 and returned to normal oxygen conditions (normoxia) at P12. The retinas were dissected at P12, P17, and P21.

#### RNA isolation, cDNA synthesis, RT-qPCR

Total RNA was extracted with the Rneasy Micro or Mini Kit (QIAGEN, Maryland, MD, USA) and reverse transcribed using the PrimeScript™ RT Master Mix (Takara) in accordance with the manufacturer’s protocol. RT-qPCR was performed on a 7500 Fast Real-Time PCR System (Thermo Fisher Scientific, Waltham, MA, USA) using THUNDERBIRD® SYBR® qPCR Mix (TOYOBO, Osaka, Japan). The thermal cycling conditions were as follows: 95°C for 20 s and 45 cycles of 95°C for 3 s and 60°C for 30 s. The primers used for RT-qPCR are shown in STAR Methods. Relative gene expression was assessed using the 2−ΔΔCt method.

#### Transfection

HUVECs and HRMECs were cultured to 40%–60% confluence and transfected for 24 h with Stealth siRNA targeting human *ATF3* (Invitrogen) and Stealth RNAi™ siRNA Negative Control (Invitrogen) or Silencer Select siRNAs targeting human *ATF3* (Ambion, Austin, TX, USA) and Silencer Select Negative Control No. 2 siRNA (Invitrogen) by using Lipofectamine™ RNAiMAX Transfection Reagent (Invitrogen) in accordance with the manufacturer’s instructions. We used siRNA #2 in Figure 8. Transfection efficiency was evaluated using RT-qPCR.

#### Overexpression of *ATF3*

The lentiviruses (LVs) for overexpressing *ATF3* (LV-CMV:: *ATF3-T2A-eGFP*) and the control LV (LV-*CMV::mCherry-T2A-eGFP*) were generated by using a Lentiviral High Titer Packaging Mix (Takara), TransIT-293 Transfection Reagent (Mirus Bio, Madison, WI, USA), and Lenti-X™ 293T Cell Line (Takara). HUVECs were infected with LV-CMV:: *ATF3* or LV-Control, respectively. GFP expression was observed to assess infection efficiency based on fluorescence microscopy. RT-qPCR and western blot were used to confirm the expression of *ATF3* mRNA and protein.

#### Western blot

Cells were harvested and lysed in RIPA buffer (50 mmol/L Tris-HCl Buffer, 150 mmol/L NaCl, 1% Nonidet P40 Substitute, 0.5% sodium deoxycholate, and 0.1% SDS) (Nacalai Tesque, Kyoto, Japan). After centrifugation, the protein concentrations of the supernatants were detected using the Pierce™ BCA Protein Assay Kits (Thermo Fisher Scientific). The proteins were resolved on SDS-PAGE gels (Thermo Fisher Scientific) and transferred onto PVDF membranes (Cytiva, Marlborough, MA, USA). After blocking in Tris-buffered saline/Tween-20 (TBST) containing 5% BSA for 1 h at room temperature, the membranes were incubated with the primary antibodies overnight at 4°C and then with the secondary antibodies for 1 h at room temperature. The immunoreactive bands were detected using the ECL™ Prime Western Blotting Detection Reagent (Cytiva). The membranes were stripped and reprobed for a loading control. The primary antibodies were anti-ATF3 (1/1000, Cell Signaling Technology) and anti-βactin (1/5000, Sigma-Aldrich). The secondary antibodies were anti-rabbit and anti-mouse IgG (1/5000, Cytiva).

#### Tube formation assay

Tube formation assays were performed in accordance with the CORNING protocol for endothelial cell tube formation. An angiogenesis assay plate was prepared in a 24-well plate using 0.289 mL of 10 mg/mL Matrigel Matrix (CORNING, Corning, NY, USA) per well. HRMECs transfected with negative control siRNAs or *ATF3* siRNAs were seeded onto the plates in triplicate and subsequently incubated for 16–18 h at 37°C in 5% CO_2_ atmosphere. Images were acquired using a Zeiss Microscope AXIO Observer.D1 (Carl Zeiss). Vascular density and vascular length density were measured using Vessel Analysis plugin for ImageJ (National Institutes of Health).

#### RNA preparation and bulk-RNA sequencing

Total RNA was extracted as previously stated in RNA isolation. A pipeline provided by Rhelixa, Inc. (Tokyo, Japan) was used to prepare cDNA libraries from RNA. The NEBNext Poly(A) mRNA Magnetic Isolation Module (New England Biolabs, Hitchin, UK) and NEBNext Ultra II Directional RNA Library Prep Kit (New England Biolabs) were used to prepare the cDNA library, and the NovaSeq 6000 system (Illumina, Yokohama, Japan) was used for 150-base paired-end read sequencing.

#### Analysis of bulk RNA-seq data

Raw FASTQ files were processed using fastp (v0.19.5)^44^ to remove adapter sequences and low-quality reads, and the filtered reads were aligned to the human reference genome (GRCh38) using STAR (v2.7.10b).^45^ Read counts were quantified using RSEM (v1.3.3)^46^ with the rsem-calculate-expression function. Differential expression analysis was performed using DESeq2 (v1.38.3).^47^

#### Single-cell library preparation and sequencing

Mouse retinal ECs were sorted using flow cytometry as previously described. The cell suspensions were loaded onto a Chromium Single Cell B Chip to generate Gel Bead-In-emulsions (GEMs) using Chromium Controller (10x Genomics, Pleasanton, CA, USA). Reverse transcription (RT) was performed in a thermal cycler. First-strand cDNA was purified from the post-GEM-RT reaction mixture using DynaBeads. The cDNA was then amplified using a thermal cycler, and the amplified cDNA product was purified using the SPRIselect Reagent Kit (Beckman Coulter, Brea, CA, USA). Then, 3ʹ gene expression libraries were constructed using Chromium Single Cell 3′ Library & Gel Bead Kit v3 (10x Genomics) in accordance with the manufacturer’s instructions. A 2100 Bioanalyzer Laptop Bundle (Agilent Technologies, Santa Clara, CA, USA) with a High Sensitivity DNA Kit (Agilent Technologies) was used to assess DNA quality and quantity. The single-cell library was sequenced using the HiSeq with DNAFORM (Illumina).

#### Analysis of single-cell RNA-seq data

Raw FASTQ files were processed using the Cell Ranger (v7.1.0) (10x Genomics) count pipeline. Reads were aligned to the mm10 genome (refdata-gex-mm10-2020-A). The output count data were corrected for ambient RNA expression using the R package SoupX (v1.6.2),^48^ and doublets were identified using the Python package scrublet (v0.2.3).^49^ The corrected count matrix was processed using the R package Seurat (v4.3.0).^50^ After removing the doublets, cells expressing fewer than 2,000 genes or more than 8,000 genes, as well as cells with over 10% mitochondrial genes or over 20% ribosomal genes, were excluded. Cell cycle phase scores were calculated using the CellCycleScoring function, and the data were normalized and scaled using the SCTransform function, regressing out the effects of cell cycle scores (S and G2M scores) and the percentage of mitochondrial and ribosomal genes. Following dimensionality reductions using the RunPCA and RunUMAP functions, clustering was performed using the FindNeighbours function with the top principal components that explain 90% of the variance and the FindClusters function. ECs with at least one UMI count of *ATF3* were recognized as *ATF3*-positive. Differential expression analysis between *ATF3*-positive and -negative ECs was performed using the FindMarkers function, and genes with an adjusted P-value < 0.05 were considered significant. GO enrichment analysis was performed using the enrichGO function of the ClusterProfiler package (v4.6.2).^51^ Protein–protein interactions were obtained from the STRING database (https://string-db.org/).^53^

### Quantification and statistical analysis

Quantitative data were collected from at least three independent experiments and are expressed as mean ± SEM. Normality was confirmed using the Shapiro–Wilk test before performing parametric tests (two-tailed unpaired Student's t-test) for parametric data and non-parametric tests (Wilcoxon’s test) for non-parametric data. The statistical details are described in the figures and in their corresponding legends for individual experiments. Significance was set at P < 0.05. JMP Pro software (v17.1.0) (SAS Institute, Cary, NC, USA) was used for statistical analysis.
