# Supplementary material for: Endothelial activating transcription factor 3 promotes angiogenesis and vascular repair in the mouse retina
Source: iScience. 2024 Dec 2;28(1):111516. doi: 10.1016/j.isci.2024.111516 (PMC11714383; doi:10.1016/j.isci.2024.111516)
Supplement: Document S1. Figures S1–S7 and Table S1 [file mmc1.pdf]

iScience, Volume *28*

Supplemental information

Endothelial activating transcription factor 3 promotes angiogenesis and vascular repair in the mouse retina

Chihiro Ueda, Susumu Sakimoto, Masahito Yoshihara, Toru Takigawa, Akihiko Shiraki, Kaito Yamaguchi, Kosuke Shiki, Nobuhiko Shiraki, Shigetaka Kitajima, Yoshiaki Kubota, Yoko Fukushima, and Kohji Nishida

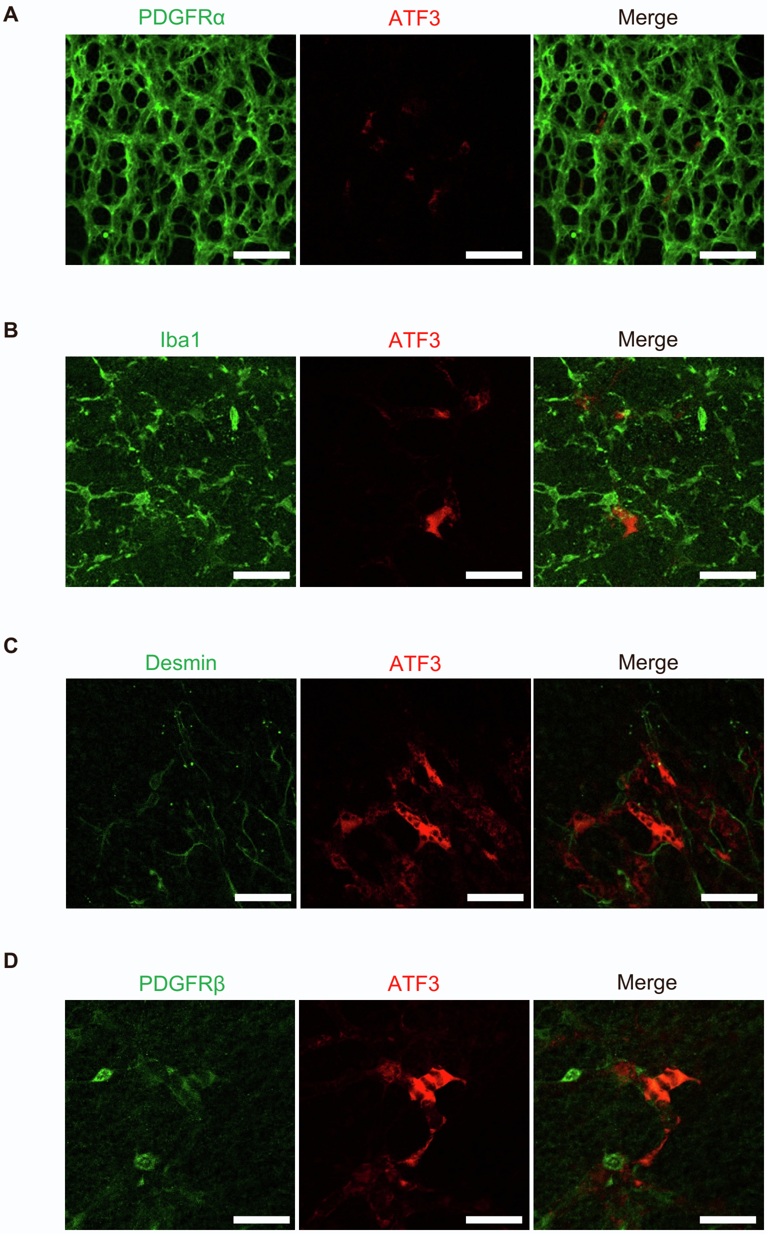

Figure S1 *ATF3* localization in the developing mouse retina, related to Figure 1.

(A) Retinal whole-mount staining of PDGFRα (green) and ATF3 (red) in WT mice at P5.

(B) Retinal whole-mount staining of Iba1 (green) and ATF3 (red) in WT mice at P5.

(C) Retinal whole-mount staining of Desmin (green) and ATF3 (red) in WT mice at P5.

(D) Retinal whole-mount staining of PDGFRβ (green) and ATF3 (red) in WT mice at P5. Scale bars: 50 µm.

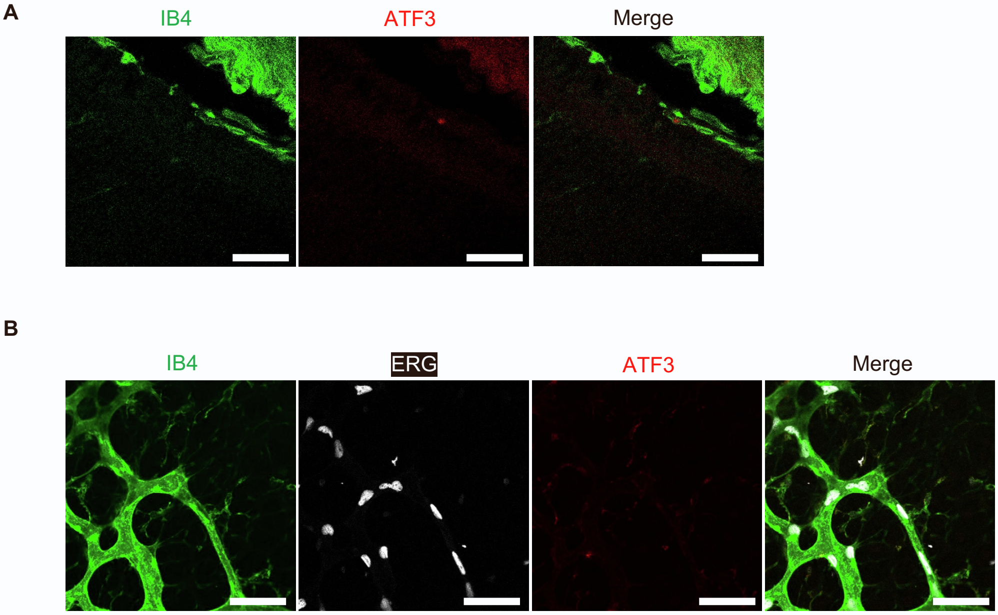

Figure S2 *ATF3* localization at vascular endothelial cells in the developing mouse retina, related to Figure 1.

(A) Retinal section staining of IB4 (green) and ATF3 (red) in WT mice at P5.

(B) Retinal whole-mount staining of IB4 (green), ERG (magenta), and ATF3 (red) in Cdh5-Cre *Atf3*$^{fl/fl}$ (*Atf3*iECKO) mice at P5.Scale bars: 50 μm.

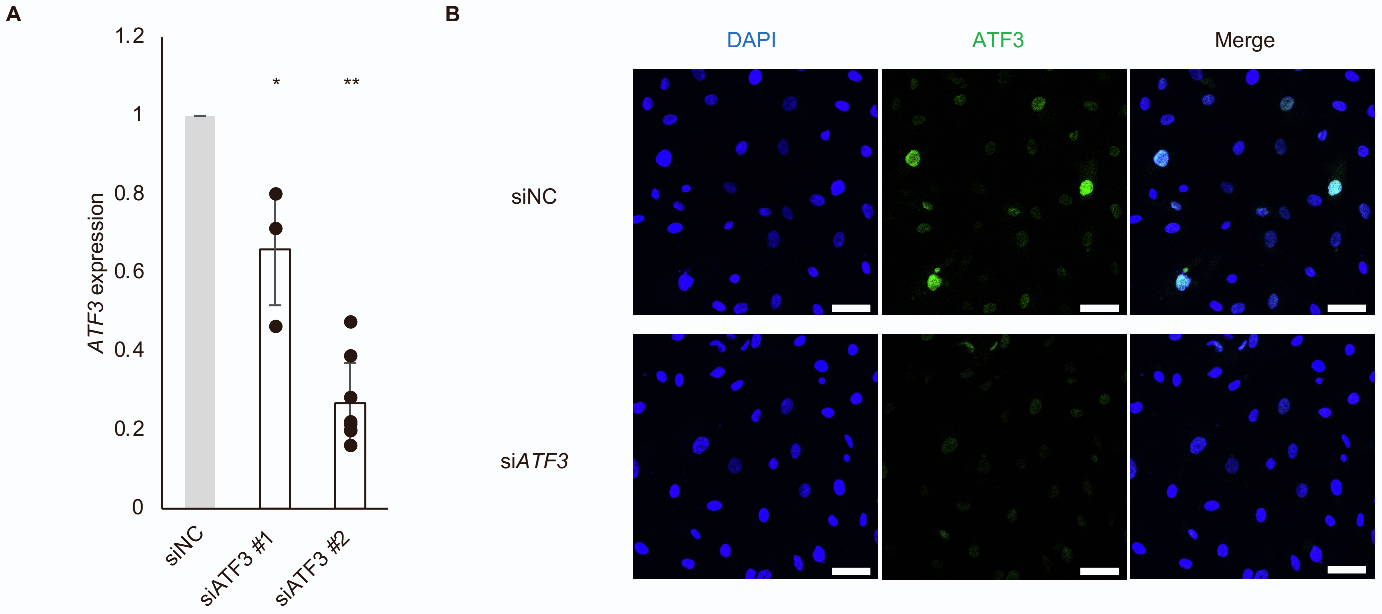

Figure S3 HRMECs transfected with siRNAs, related to Figure 2.

(A) RT-qPCR analysis of *ATF3* mRNA in HRMECs transfected with negative control siRNAs (siNC) or *ATF3* siRNAs (si*ATF3*). Error bars represent mean ± SEM. *P < 0.05, **P < 0.01.

(B) HRMECs were transfected with siNC or si*ATF3*, stimulated with VEGF (20 ng/mL) and stained with DAPI (blue) and ATF3 (green). Scale bars: 50 μm.

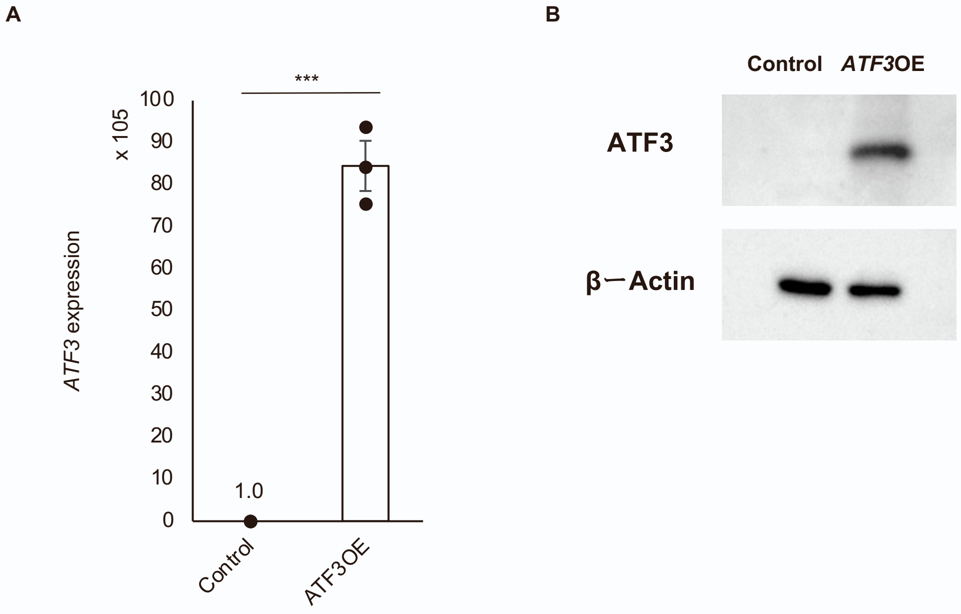

Figure S4 *ATF3*-overexpressed (*ATF3*OE) HUVECs, related to Figure 2.

(A) RT-qPCR analysis of *ATF3* mRNA in control and *ATF3*OE HUVECs. Error bars represent mean ± SEM. ***P < 0.001.

(B) Western blot analysis of ATF3 in control and *ATF3*OE HUVECs. β–Actin is shown as a loading control.

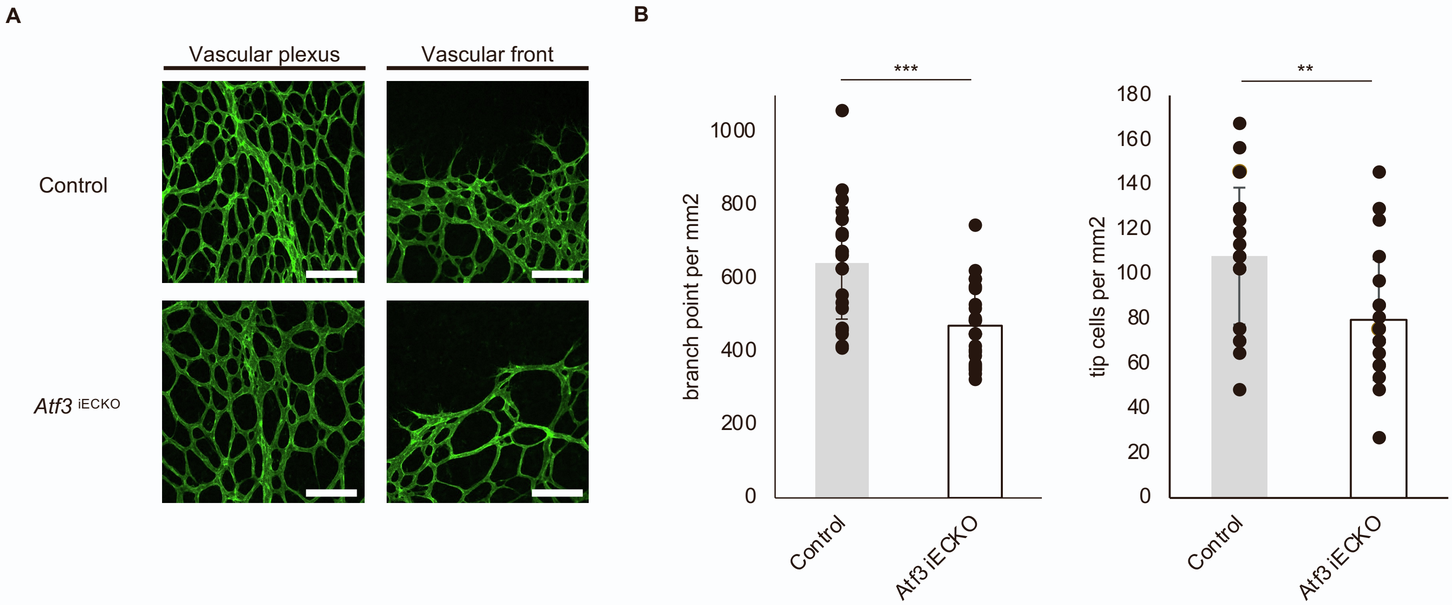

Figure S5 Endothelial *ATF3* deletion inhibits angiogenesis in developing retinal vessels, related to Figure 3.

(A) Retinal whole-mount staining of PECAM1 (green) in *Atf3*$^{fl/fl}$ (control) and Cdh5-Cre *Atf3*$^{fl/fl}$ (*Atf3*iECKO) mice at P5. Scale bars: 100 μm.

(B) Quantification of the number of branch points in the vascular plexus (left) and tip cells in the vascular front (right). Control, n = 6 eyes; *Atf3*iECKO, n = 7eyes. Error bars represent mean ± SEM. $**P < 0.01$; $***P < 0.001$.

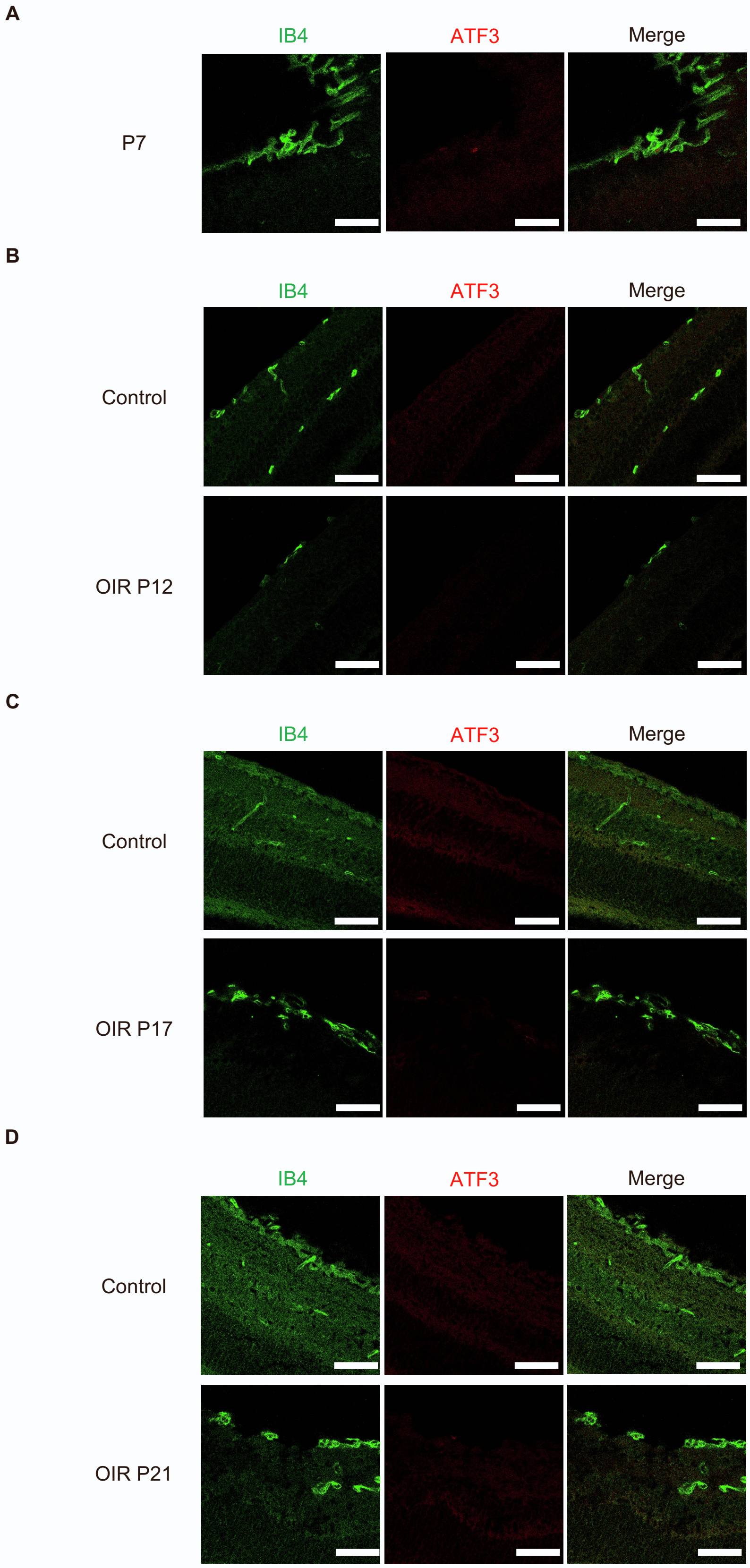

Figure S6 *ATF3* localization at endothelial cells of the OIR model, related to Figure 4.
(A-D) Retinal section staining of IB4 (green) and ATF3 (red) in WT mice at P7 (A), and control or OIR-WT mice at P12 (B), P17 (C) and P21 (D). Scale bars: 50 μm.

A

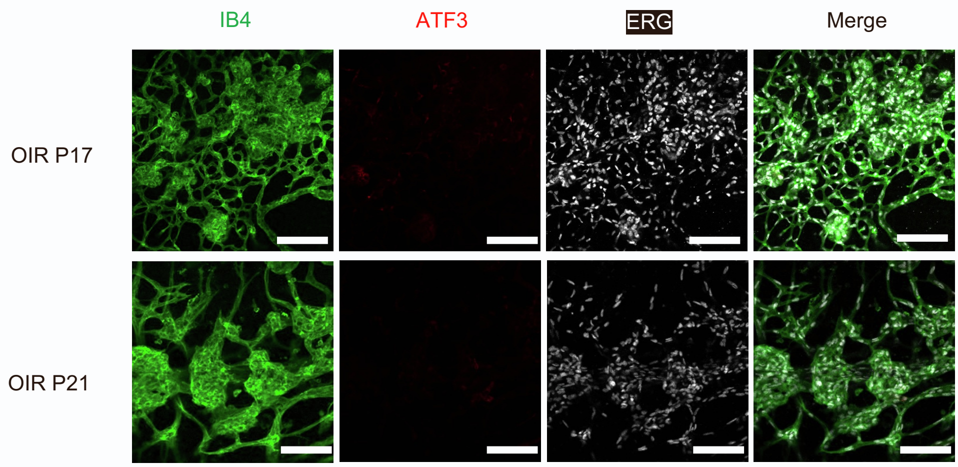

Figure S7 *ATF3* expression in NVTs of the OIR model, related to Figure 4.

(A) Retinal whole-mount staining of IB4 (green), *ATF3* (red), and ERG (white) in OIR WT mice at P17 and P21. Scale bars: 100 μm.

Table S1 List for the qPCR primers, related to STAR Methods

REAGENT or RESOURCE	SOURCE	IDENTIFIER
Oligonucleotides		
Forward qPCR primer for mouse *Gapdh*: 5′-TGGCAAAGTGGAGATTGTTGCC-3′	Sakimoto et al.[S1]	N/A
Reverse qPCR primer for mouse *Gapdh:* 5′-AAGATGGTGATGGGCTTCCCG-3′	Sakimoto et al.[S1]	N/A
Forward qPCR primer for mouse *Atf3*: 5′-CAGTCACCAAGTCTGAGGCG-3′	This paper	N/A
Reverse qPCR primer for mouse *Atf3*: 5′-GTTTCTCTGACTCTTTCTGCAGGC-3′	This paper	N/A
Forward qPCR primer for human *GAPDH*: 5′-CACCATCTTCCAGGAGCGAG-3′	This paper	N/A
Reverse qPCR primer for human *GAPDH*: 5′-GCAAATGAGCCCCAGCCTTC-3′	This paper	N/A
Forward qPCR primer for human *ATF3*: 5′-GGAATCAGTCACTGTCAGCGAC-3′	This paper	N/A
Reverse qPCR primer for human *ATF3*: 5′-CAGCTTCTCCGACTCTTTCTGC-3′	This paper	N/A
Forward qPCR primer for human *APOLD1*: 5′-GAATCCTAACTGCTTTGATGCACTT-3′	Aung et al.[S2]	N/A
Reverse qPCR primer for human *APOLD1*: 5′-ATTGGAAATGACAGGTGCCC-3′	Aung et al.[S2]	N/A
Forward qPCR primer for human *RHOJ*: 5′-TGTTTGACCACTATGCAGTTAC -3′	This paper	N/A
Reverse qPCR primer for human *RHOJ*: 5′-GTGGCCTCAGCTGGTTGTA -3′	This paper	N/A
Forward qPCR primer for human *ZEB1*: 5′-GGCCGAAAACTCCCATCTG -3′	This paper	N/A
Reverse qPCR primer for human *ZEB1*: 5′-GGAAGTTTAAGGTTGTCCATTG -3′	This paper	N/A

Forward qPCR primer for human *BCLB6*: 5′-AATCTGCGGAGCCCGTTTTA-3′	This paper	N/A
Reverse qPCR primer for human *BCLB6*: 5′-CGCAGATGTGCCACCTGTA-3′	This paper	N/A
Forward qPCR primer for human *PPP1R15A*: 5′-CTTGAGGCAGCCGGAGATAC -3′	This paper	N/A
Reverse qPCR primer for human *PPP1R15A*: 5′-GTCTGCCAGAAGCGATCACA -3′	This paper	N/A
Forward qPCR primer for human *DUSP1*: 5′-GTACTAGCGTCCCTGACAGC-3′	This paper	N/A
Reverse qPCR primer for human *DUSP1*: 5′-CCAGGTACAGAAAGGGCAGG-3′	This paper	N/A
Forward qPCR primer for human *WSB1*: 5′-CGAGAAAGAGATCGTGAGATTAC-3′	This paper	N/A
Reverse qPCR primer for human *WSB1*: 5′-ACTGTGCGATGTCCTTGTGA-3′	This paper	N/A
Forward qPCR primer for human *PLAUR*: 5′-TGCATTTCCTGTGGCTCATCA -3′	This paper	N/A
Reverse qPCR primer for human *PLAUR*: 5′-TCCTTTGGACGCCCTTCTTC-3′	This paper	N/A
Forward qPCR primer for human *SOCS3*: 5′-TTCAGCTCCAAGAGCGAGTA-3′	Kim et al.[S3]	N/A
Reverse qPCR primer for human *SOCS3*: 5′-GTCACTGCGCTCCAGTAGAA-3′	Kim et al.[S3]	N/A
Forward qPCR primer for human *IER3*: 5′-GCACTTTCCTCCAGCAACAC -3′	This paper	N/A
Reverse qPCR primer for human *IER3*: 5′-GAAGTCCCAGTTGGGGATACG -3′	This paper	N/A
Forward qPCR primer for human *FYN*: 5′-ACTATGAAGCACGGACAGAAG-3′	Yang et al.[S4]	N/A
Reverse qPCR primer for human *FYN*: 5′-TGCTGGGAATGTAACCTGTC-3′	Yang et al.[S4]	N/A
Forward qPCR primer for human *PDCH7*: 5′-AAGTACAGCAAACAGCCATTTCG -3′	This paper	N/A

Reverse qPCR primer for human *PDCH7*: 5′-GCCCGCTGTCATAGCAACTC-3′	This paper	N/A
Forward qPCR primer for human *ICAM1*: 5′-GAGGCCCCACAGACTTACAG-3′	This paper	N/A
Reverse qPCR primer for human *ICAM1*: 5′-GGTTGGGGTCAGTAGACAGC-3′	This paper	N/A

[S1] Sakimoto, S., Kidoya, H., Naito, H., Kamei, M., Sakaguchi, H., Goda, N., Fukamizu, A., Nishida, K., and Takakura, N. (2012). A role for endothelial cells in promoting the maturation of astrocytes through the apelin/APJ system in mice. Development *139*, 1327–1335. https://doi.org/10.1242/dev.072330.

[S2] Aung, H.H., Lame, M.W., Gohil, K., He, G., Denison, M.S., Rutledge, J.C., and Wilson, D.W. (2011). Comparative gene responses to collected ambient particles in vitro: endothelial responses. Physiol. Genom. *43*, 917–929. https://doi.org/10.1152/physiolgenomics.00051.2011.

[S3] Kim, T.H., Kim, K., Park, S.J., Lee, S.H., Hwang, J.W., Park, S.H., Yum, G.H., and Lee, S.H. (2012). Expression of SOCS1 and SOCS3 is altered in the nasal mucosa of patients with mild and moderate/severe persistent allergic rhinitis. Int. Arch. Allergy Immunol. *158*, 387–396. https://doi.org/10.1159/000333103.

[S4] Yang, J., Wang, Y., Zeng, Z., Qiao, L., Zhuang, L., Gao, Q., Ma, D., and Huang, X. (2017). Smad4 deletion in blood vessel endothelial cells promotes ovarian cancer metastasis. Int. J. Oncol. *50*, 1693–1700. https://doi.org/10.3892/ijo.2017.3957.
